# Quantitative Trait Locus (QTLs) Mapping for Quality Traits of Wheat Based on High Density Genetic Map Combined With Bulked Segregant Analysis RNA-seq (BSR-Seq) Indicates That the *Basic 7S Globulin* Gene Is Related to Falling Number

**DOI:** 10.3389/fpls.2020.600788

**Published:** 2020-12-10

**Authors:** Qiao Li, Zhifen Pan, Yuan Gao, Tao Li, Junjun Liang, Zijin Zhang, Haili Zhang, Guangbing Deng, Hai Long, Maoqun Yu

**Affiliations:** ^1^Chengdu Institute of Biology, Chinese Academy of Sciences, Chengdu, China; ^2^University of Chinese Academy of Sciences, Beijing, China

**Keywords:** wheat, falling number, hardness, protein, QTL, BSR, SLAF, *basic 7S globulin*

## Abstract

Numerous quantitative trait loci (QTLs) have been identified for wheat quality; however, most are confined to low-density genetic maps. In this study, based on specific-locus amplified fragment sequencing (SLAF-seq), a high-density genetic map was constructed with 193 recombinant inbred lines derived from Chuanmai 42 and Chuanmai 39. In total, 30 QTLs with phenotypic variance explained (PVE) up to 47.99% were identified for falling number (FN), grain protein content (GPC), grain hardness (GH), and starch pasting properties across three environments. Five *NAM* genes closely adjacent to *QGPC.cib-4A* probably have effects on GPC. *QGH.cib-5D* was the only one detected for GH with high PVE of 33.31–47.99% across the three environments and was assumed to be related to the nearest *pina-D1* and *pinb-D1*genes. Three QTLs were identified for FN in at least two environments, of which *QFN.cib-3D* had relatively higher PVE of 16.58–25.74%. The positive effect of *QFN.cib-3D* for high FN was verified in a double-haploid population derived from Chuanmai 42 **×** Kechengmai 4. The combination of these QTLs has a considerable effect on increasing FN. The transcript levels of *Basic 7S globulin* and *Basic 7S globulin 2* in *QFN.cib-3D* were significantly different between low FN and high FN bulks, as observed through bulk segregant RNA-seq (BSR). These QTLs and candidate genes based on the high-density genetic map would be beneficial for further understanding of the genetic mechanism of quality traits and molecular breeding of wheat.

## Introduction

Wheat (*Triticum aestivum* L.) is an important cereal crop, being one of the main sources of food for approximately 35% of the world’s population (FAOSTAT, 2013; Curtis and Halford, 2014; Liu J. et al., 2017). Currently, wheat is widely consumed and processed into bread, noodles, cakes, pasta, beer, and other products. Enhancement in the quality and yield of wheat has significant impacts on food security and human health. Improving end-use quality to meet the increasing market demand is becoming a critical topic in crop sciences (Kong et al., 2013; Amiri et al., 2018). The quality traits of wheat are controlled by many genes and are easily influenced by the environment. Quantitative trait locus (QTL) analysis and genome-wide association studies are regarded as useful tools for understanding the genetic mechanism and for identifying markers for marker-assisted selection (MAS) of quantitative traits of wheat.

Grain hardness (GH), which defined endosperm texture, is the most important determinant for wheat quality and classification (Pasha et al., 2010). Difference in GH is suggested to be due to the continuity of the protein matrix and the strength with which it physically entraps starch granules (Pomeranz and Williams, 1990). Changes in GH affect milling, baking, and other end-use applications of wheat. Hard wheat is suited for fermented products such as bread, because starch granules were liable to be broken under stronger grinding forces, and these broken starch granules with higher water-absorbing capacity and enzymatic digestion rate are beneficial for yeast growth and fermentation (Henry, 1996; Pasha et al., 2010). In contrast, for soft wheat, more intact starch granules remain during the milling process; soft wheat is usually used in the production of biscuits, cakes, pastries, and confectionaries. The major QTL for GH is located at the *Ha* loci of chromosome 5DS (Mattern, 1973; Law et al., 1978). *Pina*-*D1* and *Pinb*-*D1* genes are close to the *Ha* loci, encoding “friabilin,” which is an endosperm-specific lipid-binding protein that has been proven to largely determine the GH of wheat (Morris, 2002). However, up to 40% of the variation in GH was due to other unknown factors (Weightman et al., 2008).

Grain protein content (GPC) is another key factor determining the nutritional value and end-use quality of wheat. For example, wheat with higher GPC is suitable for making bread, pasta, and yellow alkaline Chinese noodles (Ramen); however, for white-salted Japanese noodles (Udon), wheat flour with low or moderate GPC is preferred (Distelfeld et al., 2008). In general, GPC is a complex trait controlled by multiple quantitative QTL/genes (Tsilo et al., 2010; Simons et al., 2012; Kumar et al., 2018) and largely influenced by the environment (Chope et al., 2014; Fatiukha et al., 2019). Approximately 367 QTLs identified for GPC have been mapped on all chromosomes of wheat, explaining the phenotypic variance of 0.6–66%. Most major QTLs have been detected on chromosomes 2A, 2B, 3A, 4A, 6B, 7A, and 7B (Kumar et al., 2018). *Gpc-B1* on chromosome 6BS, explaining up to 66% of the phenotypic variation for GPC (Olmos et al., 2003), was further fine mapped to an NAC transcription factor (*NAM-B1*), which affects nutrient remobilization (Uauy et al., 2006), is an important QTL, and is deployed in several breeding programs (Kumar et al., 2018).

The Hagberg falling number (FN), which reflects α-amylase activity in mature grains and simulates the rheological properties of starch in cooking, is an important grading index for wheat quality. Wheat flour with excessively low FN (LFN) related to higher α-amylase activity usually produces a sticky dough and discolored and poorly structured loaves (Zhang et al., 2014; Newberry et al., 2018). Elevated levels of α-amylase activity in mature grains are usually associated with preharvest sprouting (PHS) and late maturity α-amylase (LMA), which downgrades the grain (Munkvold et al., 2009). PHS refers to grain germination in the spikes of the mother plant before harvest especially during rainy days, which reduces not only the yield, but also nutrition and industry quality (Gubler et al., 2005; Newberry et al., 2018). LMA is a genetic defect with the characteristic of a single α-amylase isoform 1 (α*-Amy-1*) during later stages of grain development (Mares and Mrva, 2008; Newberry et al., 2018). QTLs for PHS and LMA rated by FN were mapped to almost all wheat chromosomes (Zanetti et al., 2000; Groos et al., 2002; Osa et al., 2003; Kulwal et al., 2004, 2011; Mares et al., 2005; Mori et al., 2005; Kottearachchi et al., 2006; Fofana et al., 2008; Mohan et al., 2009; Munkvold et al., 2009; Jaiswal et al., 2012; Cabral et al., 2014), of which most QTLs were distributed on chromosome 3A, 3B, 3D, 6B, 7B, 4A, and 4B and were related to plant height, grain color, and grain dormancy. However, the mechanism for PHS and LMA remains unclear, with few genes cloned.

Starch pasting properties usually receive far less attention than other quality traits, but pasting parameters measured by rapid-viscosity analysis (RVA) can simulate flour changes in the cooking process and are closely associated with Asian noodle and steamed bread quality (Zhang et al., 2005; Jin et al., 2016). The *Wx* gene located on chromosomes 7A, 4A, and 7D responsible for amylose synthesis has large effects on starch pasting properties (Nakamura et al., 1993; Hung et al., 2007; Blazek and Copeland, 2008; Jin et al., 2016). Although many QTLs for pasting properties were detected on all chromosomes (Bao et al., 2000; Kuchel et al., 2006; Zhao et al., 2009; Deng et al., 2015; Wang et al., 2017), few key genes have been cloned and characterized.

Thus, although many QTLs have been identified to determine the genetic basis of quality traits in wheat, the average distances of most genetic maps are too large, or markers are mainly distributed on partial regions of chromosomes. Therefore, rare genes were cloned based on QTL analysis, and few molecular markers can be developed for MAS in wheat breeding. Therefore, high-density genetic maps are necessary for the genetic dissection of complex quality traits of wheat.

High-throughput single-nucleotide polymorphism (SNP) genotyping plays an important role in constructing high-density genetic maps of wheat (Wang et al., 2014; Maccaferri et al., 2015). Specific-locus amplified fragment sequencing (SLAF-seq) based on restriction-site associated DNA tag sequencing (RAD-seq) was developed for economic and efficient high-throughput SNP discovery (Sun et al., 2013; Qi et al., 2014). SLAF-seq can provide abundant InDel and SNP markers and has been applied in high-density genetic map construction and candidate functional gene identification for many species in recent years (Zhang et al., 2013; Han et al., 2019; Ma et al., 2019; Sun et al., 2019; Zhuang et al., 2019; Wang et al., 2020). In addition, for wheat, as the genome sequencing has been completed, a predesign experiment in SLAF-seq can be performed to evaluate restriction enzymes and sizes of restriction fragments, which have improved the efficiency of SLAF-seq. SLAF-seq has been mainly used for the identification of desirable genes on alien chromosomes or for yield traits such as thousand seed weight of wheat; however, there has been no report on the quality traits of wheat based on SLAF-seq yet (Hu et al., 2016; Li G. et al., 2016; Li Q. et al., 2016; Yin J. L. et al., 2018).

In this study, a high-density genetic map was constructed based on recombinant inbred lines (RILs) derived from Chuanmai 42 and Chuanmai 39 through SLAF-seq, and QTLs for quality traits were identified. Chuanmai 42, with high yield and good resistance, is a backbone parent used in wheat breeding in southwest China. Chuanmai 39 has high protein content and strong gluten. The QTL and candidate genes would be beneficial for MAS in wheat breeding for quality.

## Materials and Methods

### Plant Materials and Field Trials

The RILs, including 193 lines derived from Chuanmai 42 (♀) **×** Chuanmai 39 (♂), were applied to construct genetic maps. Chuanmai 42 is a soft and red-grained wheat with high yield and resistance. Chuanmai 39 is a hard and white-grained wheat with higher gluten strength. The population was grown in Shuangliu, Sichuan Province, China, in 2016–2017 (E1) and 2017–2018 (E2), and in Shifang, Sichuan Province, China, in 2017–2018 (E3). The double-haploid (DH) population, including 376 lines derived from Kechengmai 4 (♀) **×** Chuanmai 42 (♂), was applied for QTL validation and was planted in Shifang, Sichuan Province, China, in 2018–2019. Each line was planted in 2 blocks; each block was 2 m long, with three rows 30 cm apart. Crop management was implemented according to local cultivation practices. Grains were collected separately by each block and dried naturally. Grains were milled using FOSS Cyclotec CT1093.

### Phenotyping

Grain protein content was detected by near-infrared reflectance spectroscopy on a Perten DA-7200 instrument (Perten Instruments, Huddinge, Sweden). GH was determined using a single-kernel characterization system 4100 (Perten Instruments, Springfield, IL, United States). Grains were ground to whole meal using a 1-mm-sieve Cyclotec mill (Foss Tecator AB, Höganäs, Sweden). FN was measured using Falling Number FN 1000 (Perten, Sweden). Pasting properties parameters were analyzed by Micro Visco-Amylo-Graph (A. W. Brabender Instruments, South Hackensack, NJ, United States) as follows: whole meal (15 g) was suspended in distilled water (98 mL) and 10% (m/v) AgNO_3_ (2 mL). The suspension was then heated from 50°C to 92°C at a rate of 7.5°C/min, held at 92°C for 5 min, cooled to 50°C at 7.5°C/min, and held at 50°C for 1 min; the rotation speed was 250 rpm. α-Amylase activity was determined according to Whan et al. (2014) and expressed in the Ceralpha unit per gram four as determined by Bradford assays (Bradford, 1976) on the CERALPHA extracts. All traits were measured for two replicates of each block of one line.

### SLAF Library Preparation and Genotyping

Total DNA was extracted from seedlings according to the CTAB procedure (Wang, 1997), quantified using NanoDrop 2000 (Thermo Scientific, Waltham, MA, United States), and stored at −80°C. SLAF library was prepared and genotyped according to Sun et al. (2013). The DNA digestion sites and the length and distribution of the resulting fragments were investigated using the wheat reference genome (IWGSC RefSeq v1.0^1^), and *Rsa*I was chosen as the appropriate restriction enzyme. *Oryza sativa* L. was used to control SLAF library preparation. Fragments of 464–484 bp were selected to generate paired-end reads on an Illumina HiSeq-2000 sequencing platform (Illumina, San Diego, CA, United States) at Biomarker Technologies Corporation in Beijing^2^. Then, low-quality reads (quality score B20e) were filtered out and sorted to each line according to duplex barcode sequences using SLAF_Poly.pl software (Biomarker, Beijing, China). High-quality reads were barcoded, terminal 5 bp were removed, and clean reads were mapped to the wheat reference genome (IWGSC RefSeq v1.0^1^) using SOAP software (Li et al., 2008).

Genotyping was performed using the Bayesian approach to ensure quality (Sun et al., 2013). First, *a posteriori* conditional probability was calculated using the coverage of each allele and the number of SNPs. Then, the genotyping quality score translated from the probability was used to select qualified markers for subsequent analysis. Low-quality markers for each marker and each individual were counted and the worse markers or individuals were deleted during the dynamic process. When the average genotype quality scores of all SLAF markers reached the cutoff value, the process was stopped. Only polymorphic SLAF tags having full parental homozygosity (aa **×** bb) were chosen. Then, high-quality SLAF markers were obtained for high-density linkage map construction referring to the following filter standard. First, average sequence depths should be > 2-fold for each line and > 4-fold for the parents. Second, markers with more than 90% (A and B genome) or 75% (D genome) missing data were removed. Third, markers with significant segregation distortion (*P* < 0.01) were excluded.

### Construction of a High Genetic Linkage Map

Based on the genotyping results of the 193 RILs and the wheat reference genome (IWGSC RefSeq v1.0^1^), a genetic linkage map including 21 linkage groups was constructed using HighMap software (Liu et al., 2014). The genetic distances of marker and genotypes of each RIL are listed in Supplementary Table 1. The physical positions were obtained by referring to the wheat reference genome, and the collinearity between the genetic and physical positions was measured using the Spearman correlation coefficient.

### QTL Mapping

Additive QTL mapping (individual environment) was performed with the package “R/qtl” in R using Haley–Knott regression. The significance of the mapped QTLs was determined at an experimental probability of error *P* < 0.05, using genome-wide LOD thresholds ≥ 3. The best linear unbiased predictors (BLUPs) were calculated for quality traits over different environments using SAS. The walking step was set to 1 cM. Genetic maps of chromosomes with significant QTL were drawn using MapChart version 2.1.

### QTL Validation

Quantitative trait loci *QFN.cib-3D* was selected for validation in the DH derived from Kechengmai 4 × Chuanmai 42 containing 376 lines. As shown in Supplementary Table 2, four pairs of primers (3D55982, 3D55956, 3D56098, and 3D56055) were designed for *QFN.cib-3D* based on the four flanking SLAF markers (SLAFs) (M56098, M56055, M56098, and M56055). Parents and 310 lines from DH were randomly chosen for amplification with the four pairs of primers. At least three amplifications were applied to each line. The amplified bands were sequenced by TSINGKE Biological Technology Company (Beijing, China). Then, the SNP differences in the amplified sequence were compared among parents and DH lines, to identify the effect of *QFN.cib-3D* on FN.

### Annotation of Genes Within QTL Region and Comparison With Previous Studies

Flanking SLAF markers of QTLs stable in at least two environments were used to blast against IWGSC RefSeq v1.0^3^ to obtain the physical locations and were compared to previously reported QTLs. The sequences of previously reported QTLs were obtained from GrainGenes^4^ or “iwgsc_refseqv1.0_Marker_mapping_summary_2017Mar13/” downloaded from https://urgi.versailles.inra.fr/download/iwgsc/. Genes within the QTL region were retrieved from CDS sequences in IWGSC_RefSeq_Annotations_v1.0^5^. Gene function was analyzed using UniProt, COG, GO, Swissprot, and KEGG.

### Bulked Segregant Analysis by RNA-Seq

Referring to FN in three environments, 10 lines of RILs derived from Chuanmai 42 and Chuanmai 39 with extremely high FN (HFN) and LFN from RILs were chosen for bulk segregant RNA-seq (BSR). Total RNA was extracted from grains at 35 days after flowering using the TRIzol method for each chosen line. Then, equal amounts of RNA from the 10 lines with extremely high FN were mixed as HFN bulk, and equal amounts of RNA from 10 lines with extremely low FN were mixed as LFN bulk. Two libraries including the two bulks were constructed using the NEBNext Ultra RNA Library Preparation Kit (New England Biolabs, United States) and sequenced through Illumina HiSeq^TM^ 2500 platform (Illumina, United States). Reads with adaptors or more than 10% unknown nucleotides and low-quality reads (> 50% bases with a quality score ≤ 20) were removed. The filtered reads were aligned to IWGSC RefSeq v1.0 using HISAT2.0.4.

Gene expression differential display was analyzed using the DEGseq package (version 1.18.0). The statistical significance of differentially expressed genes (DEGs) was determined using a combination of multiple tests and false discovery rate (FDR). Genes with FDR < 0.05 were classified as significant DEGs. Gene function was analyzed using UniProt, GO, KEGG, and Mapman. GO and KEGG pathway enrichment analyses were performed using GO Seq 2.12 and KOBAS v2.0, with FDR < 0.05.

Single nucleotide polymorphisms were obtained initially using the GATK package (Genome Analysis Toolkit, v3.2-2; Broad Institute, United States). These SNPs were then filtered based on the following criteria: (1) sequencing depth for each SNP ≥ 7; (2) SNP index of HFN and LFN is both lower than 0.3. The SNP index value was calculated using the MutMap method (Abe et al., 2012). Then, the ΔSNP index for each SNP was calculated through the following formula: ΔSNP index = —(SNP index of HFN bulk) - (SNP index of LFN bulk)—. The average value for the ΔSNP index in the corresponding window was calculated using a sliding window with a window size of 1 Mb and slides at a size of 1 kb. SNPs with ΔSNP index > 0.80 in candidate regions were considered as candidate loci related to FN.

### Statistical Analysis

Analysis of variance was conducted by the general linear model using SPSS Statistics 20. The phenotypic variance of each trait included $n\,\gamma \,\sigma_{G}^{2}+\gamma \,\sigma_{G\,E}^{2}+\sigma_{e}^{2}$, where *n* is the number of environments, *r* is the number of blocks, $\sigma_{G}^{2}$ is the genetic variance, $\sigma_{G\,E}^{2}$ is the genotype × environment variance, and $\sigma_{e}^{2}$ is the error variance. The entry-based broad sense heritability (*H*^2^) was measured by *H*^2^ = $\sigma_{G}^{2}/\left(\sigma_{G}^{2}+\sigma_{G\,E}^{2}/n+\sigma_{e}^{2}/n\,\gamma \right)$. The significance test and Pearson correlation were analyzed by SPSS Statistics 20.

## Results

### Phenotypic Variation of Quality Traits

Grain protein content, GH, FN, and starch pasting parameters exhibited continuous variation in each environment, of which GH showed a bimodal distribution (Supplementary Figure 1). GPC, GH, and FN of the male parent Chuanmai 39 were consistently and significantly higher than those of the female parent Chuanmai 42 in all three environments (Table 1). Chuanmai 42 was characterized by higher peak viscosity (PV), through viscosity (TV), and final viscosity (FV), but no significant difference in starch pasting parameters was found between parents (Table 1). The RILs exhibited high variations among these quality traits, of which GH showed the highest coefficient of variation (CV) across the three environments, from 40.62% (E1) to 42.37% (E3), and peak time (PT) showed the lowest CV ranging from 4.36% (E2) to 5.21% (E3) (Table 1).

**TABLE 1:** Quality traits of parents and RILs in different environments.

**Trait**	**Env**	**Parent (mean ± SD)**		**RILs**		
		**Chuanmai 39**	**Chuanmai 42**	**Mean ± SD**	**Range**	**CV**
GPC (%)*	E1	15.190.39	12.110.45	15.861.63	12.55–20.30	10.28
	E2	15.820.00	12.470.52	13.541.31	10.40–17.12	9.68
	E3	15.730.55	11.820.41	12.711.21	9.72–16.74	9.52
FN (s)*	E1	398.2920.11	282.1515.18	516.16139.96	104–969	27.12
	E2	437.0010.00	293.0038.18	333.92111.84	119–756	33.49
	E3	401.5013.44	301.0019.80	287.0485.32	96–466	29.72
GH*	E1	70.310.51	26.310.34	54.4522.11	19.07–90.23	40.62
	E2	75.610.44	28.490.32	51.4421.80	19.30–88.02	42.37
	E3	73.200.39	26.000.33	52.7022.36	19.55–91.89	42.43
BD (BU)	E1	116.6115.87	165.7816.11	181.1667.18	41–386	37.08
	E2	119.5016.26	148.0015.56	149.2040.19	59–299	26.94
	E3	126.0015.56	201.0016.24	161.2552.38	36–314	32.48
PT (min)	E1	5.320.15	5.320.12	5.380.24	5.07–7.93	4.46
	E2	5.590.12	5.520.07	5.500.24	5.23–8.10	4.36
	E3	5.620.12	5.670.14	5.570.29	5.13–8.27	5.21
PV (BU)	E1	664.5315.78	823.8921.14	832.8450.27	701–1,009	6.04
	E2	752.0016.97	846.5020.18	826.5072.87	562–965	8.82
	E3	647.0018.54	888.0025.00	842.5374.26	527–998	8.81
SB (BU)	E1	327.7538.14	376.2032.47	411.62130.67	29–591	23.93
	E2	366.5025.19	359.0024.12	351.8268.58	102–639	19.49
	E3	323.5024.72	433.0027.00	353.0169.72	140–538	19.75
TV (BU)	E1	547.9122.57	691.1325.81	651.6763.54	508–797	9.75
	E2	632.5033.21	768.0030.20	676.8279.90	352–816	11.81
	E3	521.0025.45	687.0025.87	681.2884.95	385–861	12.47
FV (BU)	E1	875.6619.79	1,034.3121.46	1,080.2487.58	537–1,218	8.11
	E2	999.0020.57	1,057.5028.31	1,027.8185.96	700–1,197	8.36
	E3	844.5025.35	1,120.0030.21	1,034.2876.44	712–1,230	7.39

*BD, break down value; PT, peak time; SB, setback value. ^∗^Significant difference in quality traits between parents.*

The broad-sense heritability (*H*^2^) values of FN, GH, and GPC were 0.78, 0.71, and 0.75, respectively, which indicated that phenotypic variations were mainly due to genetic differences, although genotype, environment, and genotype × environment (*G* × *E*) interaction had significant effects on all traits (Supplementary Table 3).

The Pearson correlation was analyzed among quality traits (Supplementary Figure 2). Higher correlation was detected among pasting parameters especially PV, TV, and FV (*r* = 0.482^∗∗^–0.975^∗∗^), in the three environments. In terms of the correlation between pasting parameters and GPC, FN, and GH, higher and significant correlations were found in E1 as follows: *r* = 0.401^∗∗^–0.697^∗∗^, *r* = 0.519^∗∗^–0.722^∗∗^, and *r* = 0.254^∗∗^–0.559^∗∗^. The correlation between pasting properties and GPC and FN was slightly higher than that between pasting properties and GH. Among these pasting properties, PT and FV were more relevant to GPC, FN, and GH with higher correlation coefficients (*r* = 0.551^∗∗^–0.697^∗∗^). In terms of the correlation among GH, FN, and GPC, a higher and significant correlation was found in E1. For E1, a relatively higher correlation was detected between GH and FN (*r* = 0.722^∗∗^) than between FN and GPC (*r* = 0.501^∗∗^) and between GPC and GH (*r* = 0.431^∗∗^).

### SLAF-seq and Genotyping of RILs Population

In total, 233.90 Gb of raw data, including 1,169.49 M pair-end reads, were obtained by high-throughput sequencing of the SLAF libraries. The average Q30 ratio and guanine–cytosine content for parents and progenies were 90.61% and 43.70%, respectively (Supplementary Table 4). A total of 397,199 high-quality SLAFs harboring 4,448,250 SNPs were detected. After removing markers lacking polymorphisms between parents and low-quality markers with average sequence depths less than 4-fold, 114,140 SNPs were retained and classified into eight segregation patterns (aa × bb, ab × cc, cc × ab, ef × eg, hk × hk, lm × ll, nn × np). Only aa **×** bb pattern including 88,234 SNPs was used for construction of the genetic map.

### Construction of a High-Density Genetic Map Based on SLAFs

The detected 88,234 SNPs were further filtered, and 13,599 SLAFs were retained, of which 12,674 SLAFs (93.20%) were assigned to 21 chromosomes compared with the reference genome IWGSC RefSeq v1.0. The genetic map spans 2,859.94 cM with an average interval of 0.23 cM (Supplementary Table 5). The average distance between markers of each chromosome was lower than 1 cM, except for chromosomes 1D and 6D (Supplementary Table 5). The average marker densities of chromosomes 1B and 1D was highest (0.05 cM) and lowest (1.11 cM), respectively (Supplementary Table 5). A total of 93 markers (0.73%) showed segregation distortion at a significance level of *P* < 0.01. These 93 distorted markers were distributed on chromosomes 6B (50), 5B (19), 2B (9), 2A (8), 2D (5), and 6A (2).

The A genome included 3,759 SLAFs (29.66%), covering 859.89 cM with an average marker density of 0.30 cM. The B genome contained 6,887 SLAFs (53.96%) and spanned 1,022.87 cM with an average interval of 0.20 cM, and the D genome included 2,386 SLAFs (15.89%) covering 977.18 cM with an average interval of 0.66 cM (Supplementary Table 5). SLAFs are concentratedly distributed on the B genome, and unfortunately, the polymorphisms detected in the D genome were still relatively low.

**FIGURE 1:**
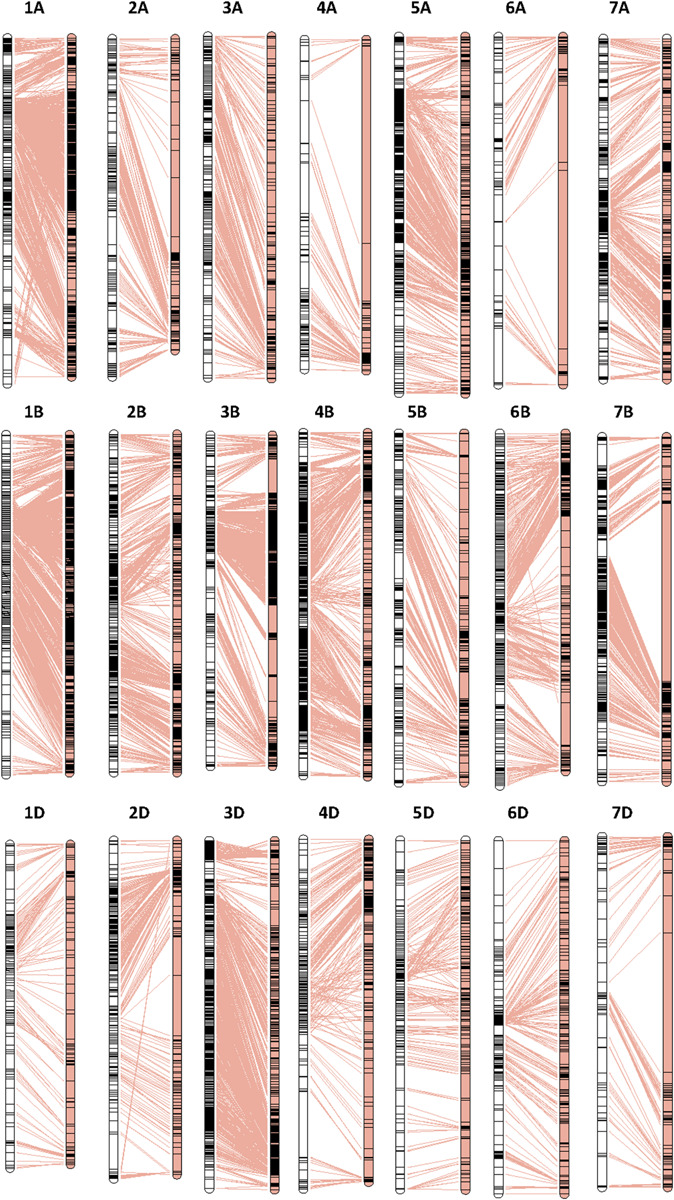
High-density genetic map construction based on SLAFs and collinearity analysis of the SLAFs between the genetic map and the physical map. The left and right panels represent the genetic map and the physical map, respectively.

Collinearity analysis results of the SLAFs between the genetic map and the physical map of wheat are shown in Figure 1 and Supplementary Figure 3. The average Spearman correlation coefficient of 21 chromosomes was 0.96 (Supplementary Table 6). This indicated that the genetic map constructed by SLAFs had a sufficient coverage over the wheat genome, and the majority of SLAFs on the linkage map were of the same order as those on the corresponding chromosomes of the physical map of the wheat genome.

### Additive QTL Analysis for Quality Traits

**FIGURE 2:**
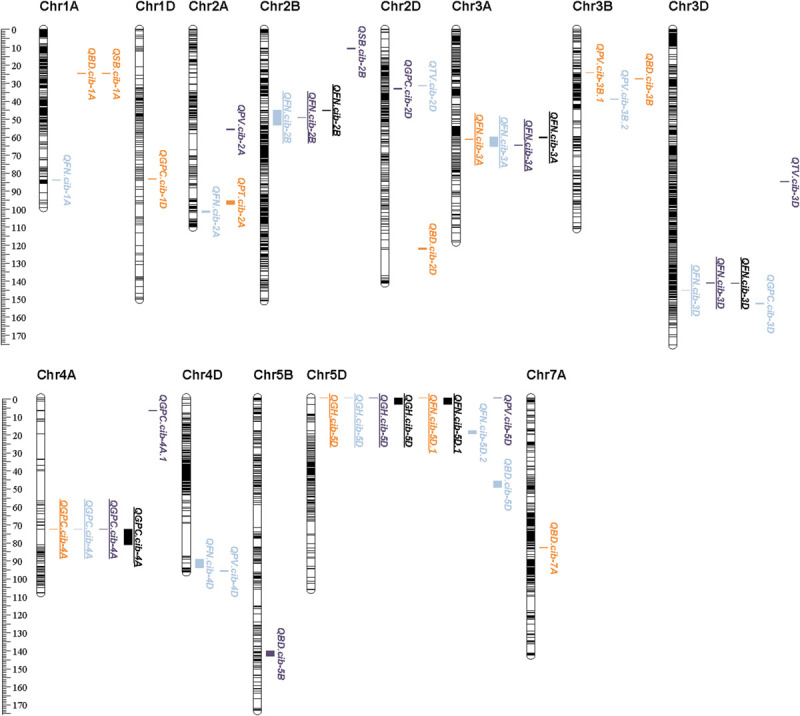
Distribution of QTLs on chromosomes. Orange-marked QTLs were detected in E1, blue ones were detected in E2, purple ones were detected in E3, and black ones were detected by BLUP. Underlined QTLs denote those that could be detected in at least two environments or by BLUP.

A total of 30 QTLs were identified for FN, GPC, GH, and starch pasting properties (Table 2, Supplementary Table 7, and Figure 2). Most QTLs were distributed on chromosome 5D (5), followed by 3D (3), 3B (3), 2D (3), 2A (3), and 1A (3) (Table 2, Supplementary Table 7, and Figure 2). Five of the 30 QTLs were stable in at least two environments also detected by the BLUPs (Table 2 and Figure 2). The average phenotypic variance explained (PVE) by individuals of the 5 QTLs varied from 4.32% to 25.74% (Table 2).

**TABLE 2:** Additive QTL characterization for quality traits in RILs of wheat across different environments*.

**QTL**	**Chr**	**Position (cM)**	**Physical distance (bp)**	**Marker interval**	**LOD**	**Additive effect**	**PVE (%)**	**Env.**	**Previously reported QTL or genes**
**FN**									
*QFN.cib-3A*	3A	60.93–61.39	665,089,892–665,719,134	M40843–M40845	4.58	−51.33 to −51.34	12.94–12.95	E1	*Vp-1A* (Yang et al., 2007)
		59.90–65.16	659,709,535–669,003,077	M40796–M40794	3.21–3.39	−28.77 to −29.81	10.95–11.75	E2	
		64.23–64.70	659,709,535–669,003,077	M40918–M40793	5.23–5.26	−38.8 to −38.82	11.59–11.60	E3	
		59.90–60.20	660,715,550–665,099,094	M40796–M40844	5.79	8.47	18.30	BLUP	
*QFN.cib-2B*	2B	44.77–53.10	144,970,701–226,440,533	M29420–M30439	3.07–3.17	−19.67 to −21.27	5.12–5.98	E2	*QPhs.cnl-2B.1* (WMC474) (Munkvold et al., 2009), ABA receptor, genes involved in calcium signaling (Somyong et al., 2011; Somyong et al., 2014)
		48.93	165,935,653–185,743,586	M29775–M29669	2.81	−23.69	4.32	E3	
		44.77–45.08	145,086,838–152,515,245	M29424–M29549	3.87	−14.94	5.53	BLUP	
*QFN.cib-3D*	3D	144.87–145.13	569,960,720–575,258,758	M56032–M56100	13.89–14.71	43.03–44.12	24.48–25.74	E2	*Tamyb10-D1*(R gene) (Yang et al., 2007), Xwmc533–Xwmc552 (Fofana et al., 2008), Xgwm314 –Xcfd9 (Groos et al., 2002)
		140.72–141.24	562,182,150–566,595,891	M55934–M55975	12.96–13.40	56.0–56.63	24.16–24.69	E3	
		140.98–141.24	563,227,642–566,551,688	M55982–M55956	10.67	25.57	16.58	BLUP	
**GH**									
*QGH.cib-5D*	5D	0	144,933–3,154,627	M85128–M85140	26.96	−15.62	47.99	E1	*Pin a* (Xmta 9) (Igrejas et al., 2002), *Pin b* (Xmta 10) (Igrejas et al., 2002)
					14.60	−13.17	33.31	E2	
					16.87	−14.39	41.95	E3	
		0–3.62	144,933–4,897,157	M85128–M85146	27.25	−14.14	44.31	BLUP	
**GPC**									
*QGPC.cib-4A*	4A	72.98	691,534,098–692,487,644	M57887–M57882	3.00	−0.38	5.11	E1	
		72.98			3.00	−0.35	7.93	E2	
		72.98			3.45	−0.35	6.81	E3	
		72.98–81.71	692,012,332–703,166,613	M57885–M57916	6.60	−0.26	12.24	BLUP	

* *QFN.cib-2B* was identified as a stable QTL because it was detected in two environments and by BLUP, although the LOD value in E3 was slightly lower than 3.

For FN, the number of QTLs (8) and major or stable QTLs (3) detected were much higher than other quality traits (Table 2 and Supplementary Table 7). All QTLs conditioned higher FN value through Chuanmai 39 alleles except *QFN.cib-3D*, with PVE ranging from 2.85 to 25.74% (Table 2 and Supplementary Table 7). Three QTLs on chromosomes 3A, 2B, and 3D could be detected in two environments and by BLUP, of which QTL *qFN-3D.1* and *qFN-3D.2* had the highest PVE of 16.58%–25.74% with additive effects ranging from 25.57 to 56.63 s (Table 2).

Five QTLs were detected for GPC, of which only *QGPC.cib-4A* located on chromosome 4A was detected in all three environments and by BLUP, explaining 5.11–12.24% phenotypic variations (Table 2 and Supplementary Table 7). All these QTLs resulting in higher GPC content were contributed by Chuanmai 39 alleles (Table 2 and Supplementary Table 7).

Only one QTL located on the ends of chromosome 5D (*QGH.cib-5D*) was detected for GH with PVE reaching 33.31–47.99% (Table 2). This QTL contributing to harder grains with a hardness index increase by 13.17–15.62 was derived from Chuanmai 39 alleles and was stable in all three environments and detected by BLUP (Table 2).

In total, 16 QTLs with a PVE of 1.89-17.38% were detected for starch pasting parameters except FV, including 5, 2, 1, 6, and 2 QTLs for PV, TV, PT, BD, and SB, respectively, but no one stable QTL was detected in at least two environments (Supplementary Table 7).

A QTL cluster on chromosome 4D was simultaneously identified for FN (*QFN.cib-4D*) and PV (*QPV.cib-4D*), with PVEs of 2.85–3.45% and 6.39–6.84% (Table 2, Supplementary Table 7 and Figure 2). Another QTL cluster located on chromosome 5D, including *QFN.cib-5D.1*, *QGH.cib-5D*, and *QPV.cib-5D*, was simultaneously identified for FN, GH, and PV, explaining 14.31–22.93%, 33.31–47.99%, and 6.78% of phenotypic variance, respectively (Table 2, Supplementary Table 7, and Figure 2).

### Effects of QTL Combination on FN

**FIGURE 3:**
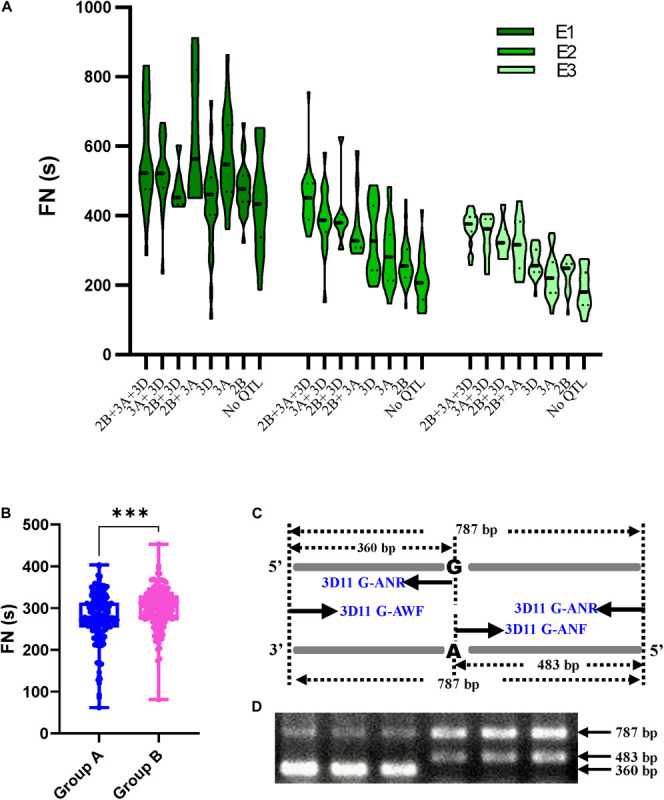
The effects of major QTLs for FN and marker development. **(A)** Effects of QTL combinations on FN of RILs in different environments shown by violin plot. **(B)** The effects of *QFN.cib-3D* on FN in RILs derived from Chuanmai 42 × Kechengmai 4. Group A denotes alleles that are the same as those of Kechengmai 4 and Chuanmai 39; group B denotes alleles that are the same as those in Chuanmai 42. **(C)** The principle of PCR-CTPP for *QFN.cib-3D*. “A” and “G” denote the SNPs in *QFN.cib-3D*. Four primers 3D11G-ANF, 3D11G-ANR, 3D11G-AWF, and 3D11G-AWR were applied in PCR. **(D)** Two patterns of PCR-CTPP amplification in RILs derived from Chuanmai 42 × Kechengmai 4. ^∗∗∗^Significant difference (*p* < 0.001) in FN between group A and group B.

To determine the combination of three major or stable QTLs (*QFN.cib-3A*, *QFN.cib-2B*, and *QFN.cib-3D*) on FN, the RILs were grouped into eight genotypes by using the flanking markers for each QTL (Figure 3A). Each genotype represents one QTL combination, which refers to a group of alleles from different loci that are inherited from parents and expressed in the progeny. Genotypes containing fewer than three lines were not analyzed.

Although there were some conflicts in E1, the FN of lines with all the three QTLs (*QFN.cib-3A* + *QFN.cib-2B* + *QFN.cib-3D*) was the highest, ranging from 375–583 s in different environments, followed by *QFN.cib-3A* + *QFN.cib-3D*, *QFN.cib-2B* + *QFN.cib-3D*, *QFN.cib-2B* + *QFN.cib-3A*, *QFN.cib-3D*, *QFN.cib-3A, QFN.cib-2B*, and null (Figure 3A and Supplementary Table 8). The null genotype without any detected QTLs showed the lowest FN of 194–442 s (Supplementary Table 8). Compared with the genotype with no QTL, the FN of *QFN.cib-3A* + *QFN.cib-2B* + *QFN.cib-3D* increased by 31.87%, 109.09%, and 93.28% in E1, E2, and E3, respectively (Supplementary Table 8). This indicated that FN was positively related to the number of QTLs, and *QFN.cib-3D* had larger effects on FN increase.

### QTL Effect Confirmation and a PCR-CTPP Marker Development

The effect of *QFN.cib-3D* was also verified in the DH population containing 376 lines derived from Kechengmai 4 **×** Chuanmai 42. The parent lines were polymorphic for four pairs of primers (Supplementary Table 2) designed for the four flanking SLAFs of these QTLs, and the RILs were classified into two groups through amplification and sequencing. A total of 310 lines were randomly chosen for *QFN.cib-3D* validation, and the four primers (3D56098, 3D56055, 3D55982, 3D55956) were closely linked in RILs derived from Chuanmai42 and Chuanmai39. Of these, 147 lines with the same alleles as Kechengmai 4 or Chuanmai 39 displayed significantly lower FN (277.03 ± 59.62 s) than the other lines (300.57 ± 45.94 s) with the same allele as Chuanmai 42 (Figure 3B). This indicated that for *QFN.cib-3D*, the alleles from Chuanmai 42 had positive effects on maintaining HFN.

A polymerase chain reaction with a confronting two-pair primers (PCR-CTPP) marker was designed for *QFN.cib-3D* to detect its presence or absence. The same allele as Chuanmai 42 was amplified with two DNA fragments of 483 and 787 bp, whereas the same allele as Kechengmai 4 or Chuanmai 39 was amplified with 360 and 787 bp (Figures 3C,D). The marker profile was in accordance with the sequencing results.

### Identification of SNPs and Candidate Genes for FN Based on BSR

**FIGURE 4:**
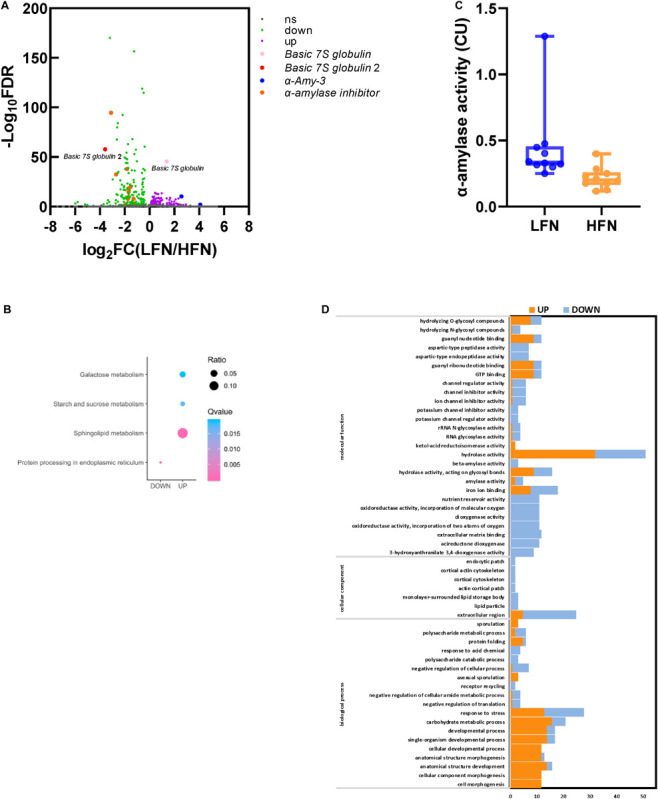
DEGs between the LFN and HFN bulk and α-amylase activity analysis. **(A)** Volcano plot of DEGs; ns represents no significant DEGs (FDR > 0.05). **(B)** KEGG enrichment analysis of DEGs. **(C)** GO enrichment analysis of DEGs. **(D)** α-Amylase activity analysis between 10 lines of the LFN and HFN bulk.

The RNA-seq result was successfully submitted to the SRA database of NCBI (accession no. PRJNA661989). In total, 80.17 and 93.94 million clean reads were obtained from the LFN and HFN bulks. The total mapped rates to IWGSC RefSeq v1.0 for the LFN and HFN bulk were 84.16 and 80.29%, respectively (Supplementary Table 9). In total, 282 significant DEGs were screened, including 170 downregulated genes and 112 upregulated genes by LFN/HFN bulk (Figure 4A). Among these genes, 10 genes were enriched in four pathways, including protein processing in endoplasmic reticulum, sphingolipid metabolism, starch and sucrose metabolism, and galactose metabolism based on KEGG enrichment analysis (Figure 4B). Through GO enrichment, 130 of these genes were enriched in 18 biological processes, 7 cellular components, and 27 molecular functions (Figure 4C). These DEGs comprised α*-Amy-3* and α*-amylase inhibitor* (Figure 4A), which is consistent with the variation in α-amylase activity between HFN and LFN bulk (Figure 4D).

A total of 117,664 SNPs and 5,993 InDels were obtained between the HFN and LFN bulks (Supplementary Figure 4). Under the threshold of ΔSNP/InDel index > 0.80, 27 candidate regions distributed on chromosomes 1B, 2A, 2B, 3A, 3B, 3D, 4A, 5B, 7A, and 7B, including 37 SNPs and InDels related to FN, were screened (Table 3 and Supplementary Figure 4). Most of the candidate regions were located on 3D (13), followed by 3A (9) (Table 3 and Supplementary Figure 4). Compared to the QTL results, two candidate regions partially overlapped with *QFN.cib-3A* and *QFN.cib-3D*, respectively, suggesting that there were genes controlling FN in the two regions. They were located on chromosome 3A (656618001–660197000, 668527001–669767000) including three SNPs and 3D (565077001–566487000) including 1 SNP. By searching *QFN.cib-3A* and *QFN.cib-3D*, two genes annotated as *Basic 7S globulin* and *Basic 7S globulin 2* annotated in *QFN.cib-3D* were detected in 282 significant DEGs. The gene expression of *Basic 7S globulin* and *Basic 7S globulin 2* was significantly higher (log_2_FC^LFN/HFN^ = 1.39) and lower (log_2_FC^LFN/HFN^ = −3.57) in the LFN than HFN bulk (Figure 4A). They are probably candidate genes related to FN.

**TABLE 3:** Candidate region and position of BSR.

**Chromosome**	**Candidate region**	**SNP/InDel**					**Type**	**Annotated gene**
		**Position**	**Reference**	**Alteration**	**index_HFN**	**index_LFN**		
chr1B	98600001_100160000	1E + 08	T	C	0.14	1	Intergenic	*Peroxidase*
	472530001_474448000	4.74E + 08	G	A	1	0.14	Intergenic	*FBD-associated F-box protein, NBS-LRR-like resistance protein*
		4.74E + 08	C	T	1	0.2	Synonymous	*NBS-LRR-like resistance protein*
chr2A	557528001_559122000	5.59E + 08	C	A	1	0.17	Intergenic	*C2 and GRAM domain-containing protein, Ribonuclease H2 subunit B*
chr2B	674064001_674066000	6.74E + 08	G	A	1	0	Intergenic	*C2 and GRAM domain-containing protein, Ribonuclease H2 subunit B*
chr3A	336877001_337499000	3.37E + 08	T	C	1	0.19	Downstream	*Translation initiation factor IF-2*
	647028001_650058000	6.48E + 08	A	G	1	0.06	Downstream	*Legume-specific protein*
		6.5E + 08	G	T	1	0	Synonymous	*plasminogen activator inhibitor*
	656618001_660197000	6.57E + 08	C	T	0.19	1	Downstream	*BTB/POZ and TAZ domain protein*
		6.57E + 08	G	A	0.1	1	Downstream	
	668527001_669767000	6.7E + 08	A	G	1	0	Downstream	*RING/U-box superfamily protein*
	676830001_677830000	6.78E + 08	AT	A	0.07	1	Intergenic	*MADS-box transcription factor, Chaperone protein dnaJ*
	681935001_684306000	6.83E + 08	G	A	0.08	1	Non-synonymous	*Glycosyltransferase family exostosin protein*
		6.83E + 08	G	C	0.09	1	Non-synonymous	
chr3B	366553001_367488000	3.67E + 08	A	AT	0.14	1	Intergenic	*Protein transport protein Sec61 beta subunit, Protein CHUP1*
	771397001_772397000	7.72E + 08	GGCC	G	1	0.14	Non-frameshift deletion	*Voltage-dependent L-type calcium channel subunit*
chr3D	476905001_478135000	4.78E + 08	A	G	1	0.11	Synonymous	*Transcription initiation factor TFIID subunit 12*
	504737001_509082000	5.06E + 08	C	T	1	0.08	Intronic	*DUF1685 family protein*
		5.07E + 08	A	G	1	0.07	Upstream	*Beta-glucosidase*
	504999001_505999000	5.06E + 08	CAG	C	1	0.14	Intronic	*DUF1685 family protein*
	516692001_517008000	5.17E + 08	A	G	1	0.11	Downstream	*2-Oxoglutarate (2OG) and Fe(II)-dependent oxygenase superfamily protein*
	520654001_520863000	5.21E + 08	A	G	1	0.07	Non-synonymous	*GPI transamidase component PIG-S*
	525977001_529347000	5.28E + 08	T	C	1	0	Synonymous	*Subtilisin-like protease*
	544416001_545650000	5.45E + 08	G	C	1	0	Iintronic	*NAC domain protein*
	550392001_552252000	5.51E + 08	A	T	1	0.07	Non-synonymous	*Alcohol dehydrogenase, putative*
		5.52E + 08	G	A	1	0	Downstream	*Auxin response factor*
	565077001_566487000	5.66E + 08	C	T	1	0	Non-synonymous	*Eukaryotic aspartyl protease family protein*
	577928001_578928000	5.79E + 08	G	C	1	0.11	Synonymous	*Amino acid permease*
		5.79E + 08	C	T	1	0	Synonymous	*Amino acid permease*
chr4A	713726001_714049000	7.14E + 08	A	T	0	1	Intergenic	*Calcium-dependent lipid-binding domain-containing protein, Receptor-like protein kinase*
chr5B	549848001_551806000	5.52E + 08	C	T	1	0.12	Downstream	*60S ribosomal protein L5*
chr7A	693565001_694565000	6.95E + 08	T	C	1	0.14	Intergenic	*DNA-3-methyladenine glycosylase, Cytochrome b561 and DOMON domain-containing protein*
		6.95E + 08	A	C	1	0.19	Intergenic	
	693565001_694565000	6.95E + 08	T	C	1	0.12	Intergenic	
		6.95E + 08	A	G	1	0.15	Intergenic	
		6.95E + 08	T	A	1	0.19	Intergenic	
chr7B	576432001_576676000	5.77E + 08	T	C	1	0	Intergenic	*60S ribosomal protein L28, Retrovirus-related pol polyprotein from transposon tnt 1-94*

## Discussion

### High-Density Genetic Map for MAS in Wheat Quality Breeding

Numerous QTL analyses have been conducted to clarify the genetic mechanism controlling complex traits of yield, quality, and resistance in wheat. However, because of the large genome size and limited genome sequence information in wheat, the one common problem is that the interval of QTL mapping is too large to further clone genes, especially for complex quality traits. Therefore, it is necessary to construct high-density genetic maps for gene identification and marker development in MAS for wheat breeding. Recently, high-density linkage map construction was facilitated by high-throughput SNP genotyping such as 55K, 90K, and 660K SNP arrays (Cui et al., 2017; Liu J. et al., 2017; Liu et al., 2018; Xu et al., 2019; Guo et al., 2020; Xin et al., 2020; Yang et al., 2020), thus increasing the accuracy and shortening the confidence interval of QTL mapping even to 0.09 cM/marker compared to those based on simple sequence repeats (SSRs) or DArTs (Jin et al., 2016). With the rapid development of next-generation high-throughput DNA sequencing, some procedures including restriction site–associated DNA tag sequencing (RAD-seq) and genotyping-by-sequencing are used in sequence-based marker development for genetic map construction, which have some advantages such as abundance, uniform distribution, and cost-effectiveness (Ganal et al., 2009; Liu H. et al., 2017).

SLAF-seq based on RAD-seq for SNP discovery and genotyping in large populations has been widely applied for high-density genetic map construction, QTL analysis, and gene cloning of extensive species such as rice (Li D. et al., 2016; Li et al., 2018; Quan et al., 2018; Zhu et al., 2018), soybean (Chen et al., 2016; Cao et al., 2017; Li et al., 2017; Kong et al., 2018; Pei et al., 2018; Yin Z. et al., 2018; Zhang et al., 2018), cotton (Zhang et al., 2016, 2017; Palanga et al., 2017; Ali et al., 2018; Keerio et al., 2018), and peanut (Hu et al., 2018; Wang et al., 2018). Some studies have investigated chromosomal localization (Li G. et al., 2016; Li Q. et al., 2016) and gene cloning for agronomic traits and resistance based on SLAF-seq of wheat (Hu et al., 2016; Yin J. L. et al., 2018). In our study, high-density genetic map construction and QTL analysis based on SLAF-seq were first applied for quality traits of wheat. Compared with most maps constructed previously by dozens or hundreds of markers such as SSRs or DArTs, the map in this study contained up to 12,674 SLAF markers; the average density of markers increased to 0.23 cM, and the region of gap > 5 cM in each chromosome was almost lower than 5%. Based on this genetic map, we identified a QTL *QGH.cib-5D* on the short arm of chromosome 5D, which is near the major QTL *Ha* loci for GH detected in previous studies (Mattern, 1973; Law et al., 1978). This suggests that the genetic map constructed by SLAFs is effective and reliable. In particular, genome sequencing of wheat has already been completed, which makes this genetic map more beneficial for discovering candidate genes for wheat quality.

### Comparison With Previous Studies and Candidate Gene Screening

#### Starch Pasting Properties Were Controlled by Many Loci With Minor Effects and Greatly Influenced by the Environment

Starch pasting properties predicted by RVA parameters were significantly associated with Asian noodle quality (Zhang et al., 2005; Wang et al., 2017). However, fewer QTL analyses for starch pasting properties have been conducted compared with those for other quality traits, and hardly any genes have been cloned, partly due to the complexity of the trait. In this study, 16 QTLs for starch pasting properties were detected, but no one was stable in at least two environments, even if SB (CV = 19.49–23.93%) and BD (CV = 26.94–37.08%) presented large variations among parents and RILs. This is consistent with the results of some previous studies that showed QTLs for pasting parameters were not stable in different environments (Mattern, 1973; Deng et al., 2015). It also suggested that starch pasting properties were complex, controlled by many loci with minor effects, and greatly influenced by the environment. Although previous studies indicated that the *Wx* gene plays an important role in starch properties (Law et al., 1978; Giroux and Morris, 1998; Deng et al., 2015), no QTLs around the *Wx* gene were detected in this study. In addition, some QTLs including *Ha* loci on chromosome 5D identified for GH, and QTLs for gluten content correlated with *Glu-B3* gene on chromosome 1B, were also detected for RVA parameters such as PV, FV, and SB (Zhang et al., 2009). These results suggest that starch pasting properties are associated with other grain quality traits and are regulated by complex genetic factors. For some QTLs explaining higher phenotypic variance, such as *QBD.cib-1A* on chromosome 1A with PVE of 17.27%–17.38%, further studies are required in more environments.

### Grain Protein Content

**FIGURE 5:**
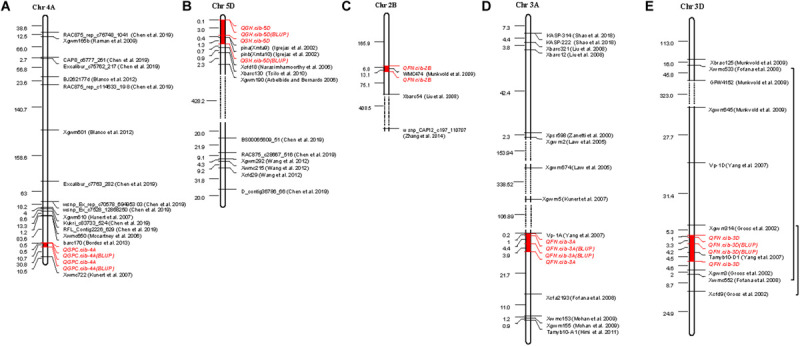
Comparison with previous QTLs or markers. Physical distance (Mbp) is listed on the left of the chromosome. Red-marked region on the chromosomes represent the QTL region detected in this study. The black bracket indicates previous QTLs overlapped with QTLs detected in this study. The dashed line represents larger area on chromosomes.

Quantitative trait locis for GPC were detected on all wheat chromosomes (Kumar et al., 2018). In this study, only one stable QTL, *QGPC.cib-4A* on chromosome 4A, was identified across all three environments (Table 2 and Figure 2). Previous studies also found GPC QTLs on chromosome 4A (Groos et al., 2003; León et al., 2006; Kunert et al., 2007; Peleg et al., 2009; Raman et al., 2009; Blanco et al., 2012; Chen et al., 2019), but they were far away from *QGPC.cib-4A* (Figure 5A). Considering the low density of makers in this area, further verification is required to confirm whether *QGPC.cib-4A* is a new QTL. The *QGPC.cib-4A* interval contained no genes, but closely adjacent to this loci, there were five consecutive genes classified into *NAC domain-containing and no apical meristem* (*NAM*) *protein*. NAC domain-containing proteins are a class of plant-specific transcription factors that play important roles in the growth and stress response. Through RNA interference, Uauy et al. (2006) showed that an NAC transcription factor, classified into *NAM protein* and encoded by *TtNAM-B1* gene, accelerates senescence and increases nutrient remobilization from leaves to developing grains, resulting in an increase in grain protein, Zn, and Fe content by more than 30% in hexaploid wheat. This suggested that these five *NAM* genes closely adjacent to *QGPC.cib-4A* have effects on GPC.

In addition, *QGPC.cib-4A* is near the gene encoding the *coatomer subunit*β’*-1* (*COPI-*β’), which is primarily responsible for intra-Golgi and Golgi to endoplasmic reticulum transport of proteins (International Rice Genome Sequencing Project, 2005). To date, there have been no reports about the relationship between *COPI-*β’and GPC, but the efficiency of protein transport may affect GPC.

### Grain Hardness

The *Ha* loci (Mattern, 1973; Law et al., 1978), a major QTL for GH, were close to the end of chromosome 5DS and contained three genes—*Pina-D1*, *Pinb-D1*, and *Gsp-1*. *Pina*-*D1* and *Pinb*-*D1* have significant effects on hardness (Giroux and Morris, 1998; Pasha et al., 2010; Chen et al., 2013; Ma et al., 2017). *Pina*-*D1* and *Pinb*-*D1* encode lipid-binding proteins and form a multiprotein complex “friabilin” bound to starch granules in the endosperm. Mutations in *Pina*-*D1* or *Pinb*-*D1* caused reduced amount of “friabilin,” thus increasing wheat GH (Giroux and Morris, 1997, 1998).

In this study, a major and stable QTL *QGH.cib-5D* was also identified at the end of chromosome 5DS with a PVE of 33.31%–47.99% in all three environments (Table 2 and Figure 2). *Pina*-*D1 and Pinb-D1* were located within this QTL detected by BLUP (Figure 5B). To analyze the relationship between *QGH.cib-5D*, *Pina*-*D1*, and *Pinb*-*D1*, *Pina-D1*, and *Pinb-D1* were amplified by the marker designed by Chen et al. (2013) and compared between Chuanmai 39 and Chuanmai 42. There was no difference in *Pinb-D1*; *Pina-D1* was missing in Chuanmai 39, which exhibited the hard phenotype. This suggested that the variance in GH was mainly due to *Pina*-*D1*. *Pina*-*D1* was further amplified and compared to the polymorphism of flanking markers (M85128 and M85140) of *QGH.cib-5D* in 147 random lines of RILs derived from Chuanmai 39 and Chuanmai 42. Specifically, if *QGH.cib-5D* was controlled by *Pina-D1*, null *Pina-D1* should correspond to “a” genotype of M85128 and M85140, the same as hard grain–type Chuanmai 39, whereas the presence of *Pina-D1* should correspond to the “b” genotype of M85128 and M85140, the same as soft grain–type Chuanmai 42. All these consistent types are shown by a transparent circle in Supplementary Figure 5A. However, the polymorphism of *Pina-D1* was not consistent with that detected by the flanking markers of *QGH.cib-5D* for 36 lines; of them, 25 lines shown in green circles did not contain *Pina-D1* but corresponded to the “b” genotype. Eleven lines shown in red circles contain *Pina-D1* but corresponded to the “a” genotype (Supplementary Figure 5A). M85128 and M85140 had advantages in screening hard grains (hardness index > 70) compared to *Pina*-*D1* (Supplementary Figure 5A). Probably genes other than *Pina*-*D1* affect GH.

There are some reports about the molecular mechanism of GH difference excluding the effect of *Pina*-*D1* and *Pinb*-*D1.* The main difference between soft and hard wheat was in adhesive strength among starch granules and the protein matrix surrounding starch granules, and proteins in soft grains are easier to separate from starch granules. Previous studies indicated that there was no relationship (Miller et al., 1984; Pomeranz et al., 1985) or some positive relationship (*r* = 0.26–0.43) between GPC and GH (Groos et al., 2004). One QTL identified for GH was close to the *GluA3* locus on chromosome 1A, which is a candidate for GPC (Arbelbide and Bernardo, 2006). However, in this study, no QTL was identified for GPC and GH simultaneously, and the correlation coefficients between GH and GPC was lower than 0.15 in the two environments (Supplementary Figure 2). This means that the difference in GH is not mainly due to GPC variance and other carbohydrates such as lipids (Morrison et al., 1989; Panozzo et al., 1993; Pasha et al., 2010) and pentosans (Hong et al., 1989; Bettge and Morris, 2000; Pasha et al., 2010).

In addition to semidwarfing genes *Rht-B1* and *Rht-D1* associated with plant height, some agronomic traits and disease resistance were found to have an effect on GH, which was reflected by major QTL on chromosome 4BS and 4DS close to the semidwarfing (gibberellin-insensitive) genes; *Reduced height (Rht)-B1* and *Rht-D1* explained up to 20 and 34% of GH variance of RILs derived from the soft × soft type (Wang et al., 2012). *Pina*-*D1* and *Pinb*-*D1* were also found to play a role in plant defense mechanisms against pathogens (Bhave and Morris, 2008; Gautier et al., 1994). This suggested that difference in GH was regulated by gibberellin and other plant hormones, and it was also related to plant defense.

In our study, the physical localization of *QGH.cib-5D* revealed 33 genes were in this region. Only 11 genes express in the grain based on gene expression data of 22 genes from WheatExp (Supplementary Figure 5B)^6^. The 11 genes include *Ethylene receptor*, *Carotenoid cleavage dioxygenase* participating abscisic acid (ABA) synthesis, *Sugar phosphatase* and *transport* genes, *Fantastic four–like protein*, *Protein kinase*, *Trypsin family protein*, and *Protein yippee-like*. *NBS-LRR disease resistance protein-like* gene was also found in *QGH.cib-5D*. These genes related to plant hormone and disease defense will also be analyzed in further studies.

### Falling Number

Falling Number is a quick evaluation method for α-amylase activity in wheat grains. Extremely high α-amylase activity with lower FN in mature wheat grain often results in downgrading quality and a sharp fall in wheat prices. PHS and LMA are two major causes of elevated α-amylase activity, but they differ in their formation mechanisms. LMA refers to high-pI α-amylase mainly synthesized during latter grain development, which seems to have little effect on grain germination and quality (Wrigley, 2006; Mares and Mrva, 2014; Newberry et al., 2018). However, the mechanism of LMA synthesis is not clear, and most QTLs for LMA were distributed on the long arm of chromosome 7B and were located near α*-Amy-2* (Mrva and Mares, 2001; Mrva et al., 2009; Barrero et al., 2013; Mares and Mrva, 2014; Mohler et al., 2014). α*-Amy-1* on chromosome 6 and α*-Amy-4* genes were also involved in LMA synthesis (Barrero et al., 2013; Mieog et al., 2017). In addition, *Rht-B1* and *Rht-D1* genes also seem to be related to LMA, because some LMA QTLs were near the *Rht-B1* and *Rht-D1* genes on chromosomes 4B and 4D, and lower LMA expression genotypes were usually semidwarf (Kunert et al., 2007; Kulwal et al., 2012; Mohler et al., 2014; Börner et al., 2017). This is probably because *Rht* and α-amylase activities were both regulated by gibberellin. In this study, no stable QTLs were identified in the above regions. However, during late grain development, it was found that the transcript level of α*-Amy-3* on chromosomes 5B and 5D of the LFN bulk was relatively higher than that of the HFN bulk through BSR results. In addition, the expression of 12 α*-amylase inhibitor* genes was significantly lower in the LFN than in the HFN bulk. It seems that α*-Amy-3* was involved in α*-*amylase activity difference among RILs, possibly resulting from an α-amylase inhibitor, although the role of α*-Amy-3* during grain development process is not clear yet.

α-Amylase activity was inhibited during grain dormancy regulated by ABA. The majority of QTLs for PHS were usually related to grain dormancy and were mainly distributed on chromosome 4A (Kato et al., 2001; Flintham et al., 2002; Mares et al., 2005; Kerfal et al., 2010), chromosome 2B (Anderson et al., 1993; Kulwal et al., 2004; Liu et al., 2008; Munkvold et al., 2009; Chao et al., 2010; Somyong et al., 2011), and chromosome 3 (Zanetti et al., 2000; Groos et al., 2002; Himi et al., 2005, 2011; Law et al., 2005; Kunert et al., 2007; Yang et al., 2007; Fofana et al., 2008; Liu et al., 2008, 2015; Mohan et al., 2009; Munkvold et al., 2009; Tan et al., 2010; Shao et al., 2018). Until now, no any candidate genes for PHS have been cloned on chromosome 4A.

In our study, eight QTLs on seven chromosomes were identified for FN (Table 2, Figure 2, and Supplementary Table 7). This indicated that FN is a complex trait and is regulated by many genes. We paid particular attention to three major or stable QTLs, including *QFN.cib-3A*, *QFN.cib-2B*, and *QFN.cib-3D. QFN.cib-2B* on chromosome 2B was detected in two environments, which partially overlapped with previous QTL *QPhs.cnl-2B.1*, which was simultaneously identified for PHS, grain dormancy, and rate of germination with the closest marker *WMC47*4 (Table 2 and Figure 5C) (Munkvold et al., 2009). Subsequently, some genes related to grain dormancy, including an ABA receptor and other genes involved in calcium signaling, were speculated to be candidates for *QPhs.cnl-2B.1* through comparative genetic analysis of rice and *Brachypodium* (Somyong et al., 2014).

We also identified a stable QTL *QFN.cib-3A* spanning 59.90–65.16 cM on chromosome 3A with a PVE of 10.95–18.30% (Table 2 and Figure 2). It could be detected in all three environments, also by BLUP over three environments and BSR (Tables 2, 3). This QTL is close to the downstream region (about 153 kb) of the *Viviparous-1* (*Vp-1*) gene (Figure 5D), which is a transcription factor that is positively correlated to grain dormancy and embryo sensitivity to ABA and inhibit α*-Amy* genes expression (Hoecker et al., 1995; Bailey et al., 1999; Yang et al., 2007). Therefore, the effect of *QFN.cib-3A* on FN may be attributed to the function of *Vp-1* gene. In addition, 95 genes were located in this QTL; three genes including *nitrate transporter NRT1-2*, *NRT1/PTR family protein 2.2*, and *cysteine proteinase inhibitor* were potential candidate genes for FN.

*QFN.cib-3D* spanning 140.72–145.13 cM on chromosome 3D was detected in two environments and by BLUP, which also explained the relatively higher phenotypic variation (16.58–25.74%) (Table 2 and Figure 2). Its effect was also validated by RILs derived from Kechengmai 4 × Chuanmai 42 (Figure 3B). *QFN.cib-3D* was located between the previous QTL for PHS and grain color, flanked by marker *Xgwm314* and *Xcfd9* (Groos et al., 2002). All these QTLs were located in another previous QTL for germination index, sprouting index and FN, flanking by *Xwmc552* and *Xwmc533* (Fofana et al., 2008; Figure 5E). This indicates the presence of important genes in this region controlling FN. A total of 149 genes were annotated in *QFN.cib-3D*. The *Tamyb10* gene, which encodes an MYB transcription factor and is a candidate fo*r R* loci of grain color, grain dormancy, and PHS, was included in *QFN.cib-3D* (Himi et al., 2005, 2011). Previous studies showed that red-grained wheat (*R-A1b*/*R-B1b*/*R-D1b*, dominant wild-type alleles) is usually more resistant to PHS than white-grained varieties (*R-A1a*/*R-B1a*/*R-D1a*, recessive mutant alleles) (Gfeller and Svejda, 1960; Debeaujon et al., 2000; Groos et al., 2002; Fofana et al., 2008; Seshu and Sorrells, 2008; Mares and Mrva, 2014) and that the *R* loci upregulate flavonoid biosynthesis involved in antioxidation, such as grain dormancy in cereal plants (Himi et al., 2005) or enhanced grain dormancy through increasing the sensitivity of embryos to ABA (Himi et al., 2002). *Tamyb10* may be candidate gene for *QFN.cib-3D*, based on that the effect of *QFN.cib-3D* on HFN was derived from the red-grained Chuanmai 42 allele.

However, no significant difference was found in transcript levels of either *Tamyb10* or *Vp-1* through BSR result (data not shown). This suggested that other genes in *QFN.cib-3A* and *QFN.cib-3D* affected FN. The transcript levels of *Basic 7S globulin* and *Basic 7S globulin 2* annotated in *QFN.cib-3D* were significantly higher and lower in the LFN than in the HFN bulk, respectively. In addition, eight other continuous genes that belong to *Basic 7S globulin 2* were in this region. They encode proteins with xylanase inhibitor domain and play roles in inhibiting cell wall degradation and radicle extension during germination. Therefore, the series of *Basic 7S globulin 2* genes could be related to α-amylase activity and could be candidates for *QFN.cib-3D* on chromosome 3D.

### QTL Clusters for Quality of Wheat

In this study, the location of *QFN.cib-4D* detected for FN and *QPV.cib-4D* detected for PV is partially overlapping (Figure 2 and Supplementary Table 7), which corresponds to the phenomenon that the measurement of FN and starch pasting properties both go through starch swelling and pasting. It suggests that some genes involved in starch synthesis and metabolism on chromosome 4D also affect FN. However, these genes have a relatively small impact on FN (PVE = 2.85–3.45%) and PV (PVE = 6.39–6.84%) and are easily disturbed by environmental deviation (Supplementary Table 7).

In addition, *QGH.cib-5D* on chromosome 5D identified for GH with PVE of 33.31–47.99% is also detected for FN (*QFN.cib-5D.1*) and PV (*QPV.cib-5D*) with PVE of 14.31–22.93% and 6.78% (Figure 2, Table 2, and Supplementary Table 7). Although *QFN.cib-5D.1* and *QPV.cib-5D* for FN and PV were detected only in single environment, it may be a major QTL that was also identified by BLUP (Supplementary Table 7). It is interesting that the expression of *Pina*-*D1* and *Pinb-D1* gene in LFN was relatively higher and lower compared to HFN bulk (data not shown). This indicated that variation of GH has large effect in FN in special environment.

A previous study also found that *QGh.caas-5D* for GH flanked by marker *Xcfd18* and *Ha* was detected in PV with a PVE of 10.4%, and *Pinb-D1b* at the *Ha* locus explained a large portion of the phenotypic variances for PV, FV, and SB, especially for pasting temperature (71.5%) (Zhang et al., 2009). Some studies have confirmed the obvious effects of GH on starch properties. Soft wheat has better starch properties than hard wheat because more intact starch granules obtained from soft grains during the mill process resulted in higher flour viscosity compared to hard grains (Rogers et al., 1993; Xu et al., 2005; León et al., 2006).

A relatively higher PVE of *QFN.cib-5D.1* means GH also has a large effect on FN in a special environment. This was also reflected by the fact that GH and FN are both related to *Rht* gene regulation by gibberellin (Tan et al., 2010; Gooding et al., 2012; Wang et al., 2012; Mohler et al., 2014; Zhang et al., 2014); therefore, some genes involved in the gibberellin response are probably candidates for *QFN.cib-5D.1.* As mentioned above, there is a pleiotropic gene or genes in *QFN.cib-5D.1* controlling FN, grain texture, starch pasting properties, and mixograph parameter (Fofana et al., 2008; Zhang et al., 2009; Tan et al., 2010; Börner et al., 2017).

## Data Availability Statement

The datasets presented in this study can be found in online repositories. The names of the repository/repositories and accession number(s) can be found in the article/Supplementary Material.

## Author Contributions

QL designed the experiment, constructed the high density genetic map, identified the QTLs and candidate genes and wrote the manuscript. ZP was involved in experimental design, instruction, and improve the manuscript. YG was involved in wheat quality determinations. TL, JL, ZZ and HZ were involved in QTLs identification. GD, HL and MY were involved in population construction and growing and improve the manuscript. All authors contributed to the article and approved the submitted version.

## Conflict of Interest

The authors declare that the research was conducted in the absence of any commercial or financial relationships that could be construed as a potential conflict of interest.

## Footnotes

1 https://wheat-urgi.versailles.inra.fr/

2 http://www.biomarker.com.cn/

3 https://wheat-urgi.versailles.inra.fr/Seq-Repository/Assemblies

4 https://wheat.pw.usda.gov/cgi-bin/GG3/browse.cgi?class=marker

5 https://wheat-urgi.versailles.inra.fr/Seq-Repository/Annotations

6 https://wheat.pw.usda.gov/WheatExp/

## Supplementary Material

Supplementary Figure 1 Frequency distribution of quality traits in the RILs of Chuanmai 42 × Chuanmai 39 in three environments.

Supplementary Figure 2 Pearson correlation between quality traits. ^∗∗^ and ^∗^means difference is significant at the 0.01 and 0.05 level, respectively.

Supplementary Figure 3 Collinearity analysis between the genetic map and the physical map of each chromosome. One grid in genetic position presented 20 cM, one grid in physical positions presented 2.0E + 08 bp. Red line presented approximate position of centromere.

Supplementary Figure 4 Distribution of all SNPs and InDels on 21 chromosomes based on ΔSNP index value and candidate regions.

Supplementary Figure 5 The relationship between *QGH.cib-5D* and *Pina-D1*, and expression of annotated genes in *QGH.cib-5D*. **(A)** The polymorphism of *Pina-D1* and flanking markers (M85128 and M85140) of *QGH.cib-5D* for 147 RILs derived from Chuanmai 42 × Chuanmai 39, the black circles denote the polymorphism of *Pina-D1* and M85128 or M85140 was consistent, the red circles denote deletion of *Pina-D1* correspond to ‘b’ genotype of M85128 and M85140, the green circles denote presence of *Pina-D1* correspond to ‘a’ genotype of M85128 and M85140. **(B)** Gene expression heat map and clustering analysis of 22 genes annotated in *QGH.cib-5D*.

Supplementary Table 1 The genetic distances of marker and genotypes of each RIL.

Supplementary Table 2 Primers applied for *QFN.cib-3D* confirmation and PCR-CTPP markers.

Supplementary Table 3 Analysis of variance and broad-sense heritability for quality traits.

Supplementary Table 4 High quality data and SNPs generated by sequencing the SLAF library.

Supplementary Table 5 Characteristics of the 21 chromosomes of the wheat genetic map.

Supplementary Table 6 Spearman correlation coefficient of genetic and physical position.

Supplementary Table 7 Additive QTL characterization for quality traits in RILs of wheat in single environment.

Supplementary Table 8 FN of eight genotypes in different environments.

Supplementary Table 9 Summary of BSA sequencing and mapping.
