# Supplementary material for: Quantitative Trait Locus (QTLs) Mapping for Quality Traits of Wheat Based on High Density Genetic Map Combined With Bulked Segregant Analysis RNA-seq (BSR-Seq) Indicates That the Basic 7S Globulin Gene Is Related to Falling Number
Source: Front Plant Sci. 2020 Dec 10;11:600788. doi: 10.3389/fpls.2020.600788 (PMC7793810; doi:10.3389/fpls.2020.600788)
Supplement: Supplementary Figure 1 — Frequency distribution of quality traits in the RILs of Chuanmai 42 × Chuanmai 39 in three environments. [file Data_Sheet_1.zip › Fig S2.DOCX]

|  |  | **GPC** | **FN** | **PT** | **PV** | **TV** | **FV** | **BD** | **SB** | **GH** |
| --- | --- | --- | --- | --- | --- | --- | --- | --- | --- | --- |
| **GPC** | **E1** | **GPC** | **0.501**** | **0.697**** | **0.654**** | **0.659**** | **0.640**** | **0.401**** | **0.506**** | **0.431**** |
|  | **E2** |  | **0.210**** | **0.045** | **-0.139** | **-0.124** | **-0.029** | **-0.036** | **0.074** | **0.149*** |
|  | **E3** |  | **0.123** | **0.149*** | **0.111** | **0.118** | **0.131** | **0.033** | **0.111** | **0.043** |
| **FN** | **E1** |  | **FN** | **0.661**** | **0.645**** | **0.602**** | **0.664**** | **0.519**** | **0.631**** | **0.722**** |
|  | **E2** |  |  | **0.286**** | **0.142** | **0.195**** | **0.202**** | **-0.093** | **0.108** | **0.109** |
|  | **E3** |  |  | **0.430**** | **0.393**** | **0.398**** | **0.404**** | **0.177*** | **0.290**** | **0.043** |
| **PT** | **E1** |  |  | **PT** | **0.972**** | **0.957**** | **0.958**** | **0.655**** | **0.796**** | **0.559**** |
|  | **E2** |  |  |  | **0.534**** | **0.494**** | **0.482**** | **0.100** | **0.192**** | **-0.038** |
|  | **E3** |  |  |  | **0.912**** | **0.865**** | **0.941**** | **0.576**** | **0.779**** | **0.009** |
| **PV** | **E1** |  |  |  | **PV** | **0.963**** | **0.975**** | **0.727**** | **0.824**** | **0.506**** |
|  | **E2** |  |  |  |  | **0.898**** | **0.737**** | **0.241**** | **0.158*** | **-0.340**** |
|  | **E3** |  |  |  |  | **0.959**** | **0.973**** | **0.599**** | **0.696**** | **-0.03** |
| **TV** | **E1** |  |  |  |  | **TV** | **0.944**** | **0.515**** | **0.709**** | **0.532**** |
|  | **E2** |  |  |  |  |  | **0.671**** | **-0.194**** | **-0.011** | **-0.297**** |
|  | **E3** |  |  |  |  |  | **0.935**** | **0.349**** | **0.538**** | **-0.046** |
| **FV** | **E1** |  |  |  |  |  | **FV** | **0.697**** | **0.903**** | **0.551**** |
|  | **E2** |  |  |  |  |  |  | **0.154*** | **0.722**** | **-0.244**** |
|  | **E3** |  |  |  |  |  |  | **0.579**** | **0.802**** | **-0.015** |
| **BD** | **E1** |  |  |  |  |  |  | **BD** | **0.814**** | **0.254**** |
|  | **E2** |  |  |  |  |  |  |  | **0.401**** | **-0.094** |
|  | **E3** |  |  |  |  |  |  |  | **0.787**** | **0.031** |
| **SB** | **E1** |  |  |  |  |  |  |  | **SB** | **0.480**** |
|  | **E2** |  |  |  |  |  |  |  |  | **-0.051** |
|  | **E3** |  |  |  |  |  |  |  |  | **0.042** |
| **GH** | **E1** |  |  |  |  |  |  |  |  | **GH** |
|  | **E2** |  |  |  |  |  |  |  |  |  |
|  | **E3** |  |  |  |  |  |  |  |  |  |

**Supplementary Figure 2 Pearson correlation between quality traits.** ** and *means difference is significant at the 0.01 and 0.05 level, respectively.
