# Supplementary material for: Quantitative Trait Locus (QTLs) Mapping for Quality Traits of Wheat Based on High Density Genetic Map Combined With Bulked Segregant Analysis RNA-seq (BSR-Seq) Indicates That the Basic 7S Globulin Gene Is Related to Falling Number
Source: Front Plant Sci. 2020 Dec 10;11:600788. doi: 10.3389/fpls.2020.600788 (PMC7793810; doi:10.3389/fpls.2020.600788)
Supplement: Supplementary Figure 1 — Frequency distribution of quality traits in the RILs of Chuanmai 42 × Chuanmai 39 in three environments. [file Data_Sheet_1.zip › Fig S3.DOCX]

**Genetic positions (cM)**

**6A**

**2A**

**1B**

**1A**

ableson **Supplementary Figure 3 Collinearity analysis between the genetic map and the physical map of each chromosome.** One grid in genetic position presented 20 cM, one grid in physical positions presented 2.0E+08 bp. Red line presented approximate position of centromere.

**Physical positions (bp)**

**Genetic positions (cM)**
