# Supplementary material for: Quantitative Trait Locus (QTLs) Mapping for Quality Traits of Wheat Based on High Density Genetic Map Combined With Bulked Segregant Analysis RNA-seq (BSR-Seq) Indicates That the Basic 7S Globulin Gene Is Related to Falling Number
Source: Front Plant Sci. 2020 Dec 10;11:600788. doi: 10.3389/fpls.2020.600788 (PMC7793810; doi:10.3389/fpls.2020.600788)
Supplement: Supplementary Figure 1 — Frequency distribution of quality traits in the RILs of Chuanmai 42 × Chuanmai 39 in three environments. [file Data_Sheet_1.zip › Fig S5.DOCX]

**A**


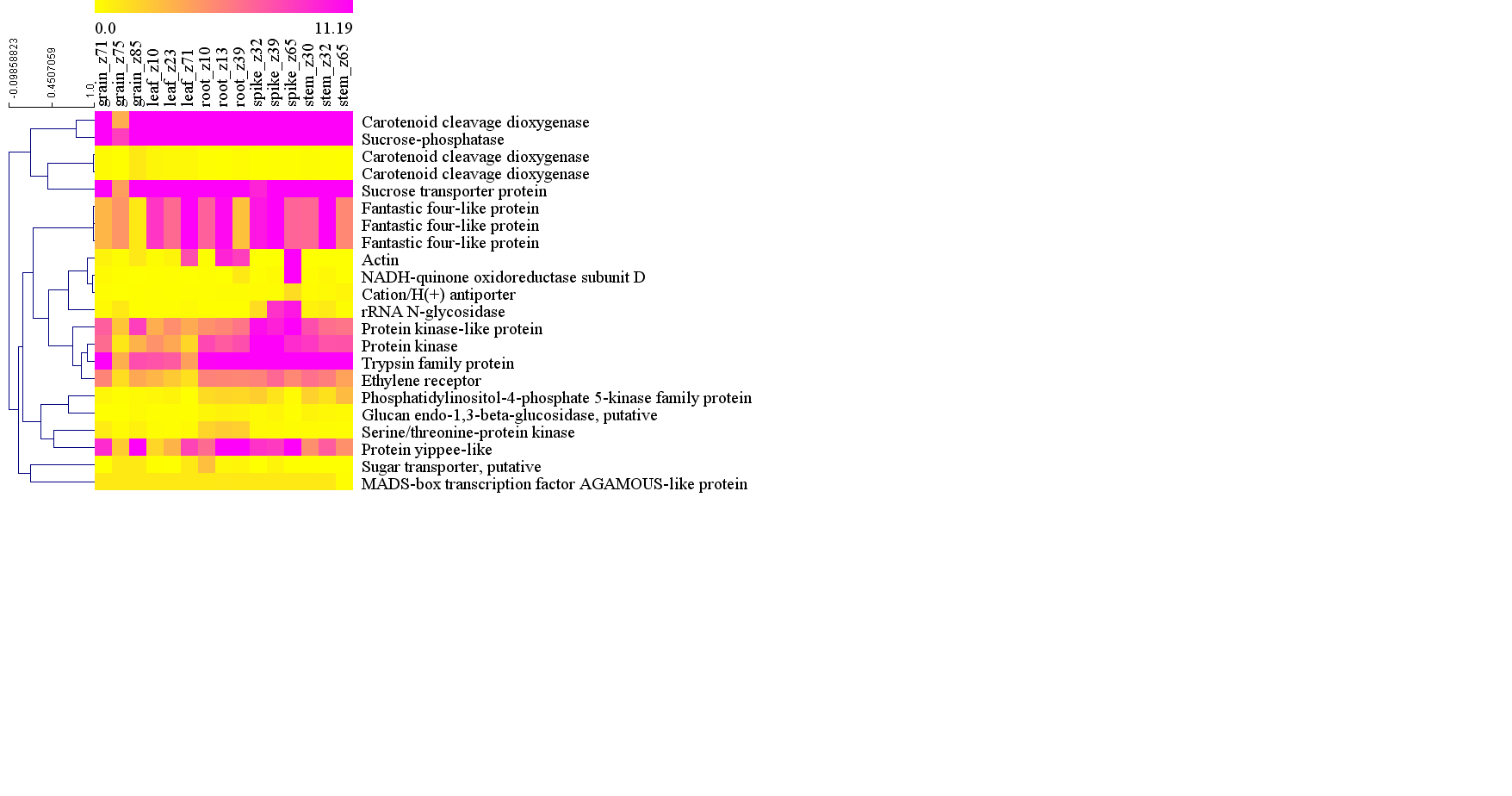


**B**

**Supplementary Figure 5 The relationship between *QGH.cib-5D* and *Pina-D1*, and expression of annotated genes in *QGH.cib-5D*. A** The polymorphism of *Pina-D1* and flanking markers (M85128 and M85140) of *QGH.cib-5D* for 147 RILs derived from Chuanmai 42 **×** Chuanmai 39, the black circles denote the polymorphism of *Pina-D1* and M85128 or M85140 was consistent, the red circles denote deletion of *Pina-D1* correspond to ‘b’ genotype of M85128 and M85140, the green circles denote presence of *Pina-D1* correspond to ‘a’ genotype of M85128 and M85140. **B** Gene expression heat map and clustering analysis of 22 genes annotated in *QGH.cib-5D*.
