# Supplementary material for: Quantitative Trait Locus (QTLs) Mapping for Quality Traits of Wheat Based on High Density Genetic Map Combined With Bulked Segregant Analysis RNA-seq (BSR-Seq) Indicates That the Basic 7S Globulin Gene Is Related to Falling Number
Source: Front Plant Sci. 2020 Dec 10;11:600788. doi: 10.3389/fpls.2020.600788 (PMC7793810; doi:10.3389/fpls.2020.600788)
Supplement: Supplementary Figure 1 — Frequency distribution of quality traits in the RILs of Chuanmai 42 × Chuanmai 39 in three environments. [file Data_Sheet_1.zip › Table S2.DOCX]

| Primer pairs | Forward and reverse PCR primer sequences | Annealing temperature (°C) |
| --- | --- | --- |
| 3D56098 | F: 5’-CACATTGCTGTCAGGTTCAC-3’ | 57℃ |
|  | R: 5’-GTTGGCGGATGCCTAATA-3’ |  |
| 3D56055 | F: 5’-GCTAAAGACACGCTCGGAT-3’ | 57℃ |
|  | R: 5’-TGAGATAAGAAGCAAACTGGAGT-3’ |  |
| 3D55982 | F: 5’-GTGCCTCATTGTTCTCCA-3’ | 57℃ |
|  | R: 5’-TCCCTGCCAACTATTGTCT-3’ |  |
| 3D55956 | F: 5’-GGCAAGAACAGCGAACTAA-3’ | 57℃ |
|  | R: 5’-ATAAGCAGGAGCAACCGA-3’ |  |
| 3D11 G-ANF | F: 5’-AAAATGAGTGAACCTATACTTTAAATGA-3’ | 58℃ |
| 3D11 G-ANR | R: 5’-AAGACTACATTGAGATGTATATAGAGGC-3’ |  |
| 3D11 G-AWF | F: 5’-TATGCGTACAATGGAAATACAAC-3’ |  |
| 3D11 G-AWR | R: 5’-CACGGCCGTATTTTTTCT-3’ |  |

**Supplementary Table 2 Primers applied for *QFN.cib-3D* confirmation and PCR-CTPP markers**
