# Supplementary material for: Quantitative Trait Locus (QTLs) Mapping for Quality Traits of Wheat Based on High Density Genetic Map Combined With Bulked Segregant Analysis RNA-seq (BSR-Seq) Indicates That the Basic 7S Globulin Gene Is Related to Falling Number
Source: Front Plant Sci. 2020 Dec 10;11:600788. doi: 10.3389/fpls.2020.600788 (PMC7793810; doi:10.3389/fpls.2020.600788)
Supplement: Supplementary Figure 1 — Frequency distribution of quality traits in the RILs of Chuanmai 42 × Chuanmai 39 in three environments. [file Data_Sheet_1.zip › Table S3.DOCX]

| Source of variation | *df* | Mean square | *F* value | *P* |
| --- | --- | --- | --- | --- |
| FN | | | | |
| Genotype | 192 | 50653.73 | 211.23 | <0.001 |
| Replicates/environment | 3 | 176.22 | 0.73 |  |
| Environments | 2 | 4693636.5 | 19572.88 | <0.001 |
| Genotype × environment | 343 | 14203.46 | 59.23 | <0.001 |
| Error | 535 | 239.8 |  |  |
| *h^2^* | 0.78 |  |  |  |
| GH | | | | |
| Genotype | 191 | 2871.13 | 118.77 | <0.001 |
| Replicates/environment | 3 | 71.96 | 2.98 |  |
| Environments | 2 | 2972.9 | 122.98 | <0.001 |
| Genotype × environment | 256 | 1126.27 | 46.59 | <0.001 |
| Error | 447 | 24.17 |  |  |
| *h^2^* | 0.71 |  |  |  |
| GPC | | | | |
| Genotype | 192 | 6.98 | 33.85 | <0.001 |
| Replicates/environment | 3 | 0.01 | 0.06 |  |
| Environments | 2 | 941.75 | 4568.6 | <0.001 |
| Genotype × environment | 359 | 2.32 | 11.19 | <0.001 |
| Error | 551 | 2.31 |  |  |
| *h^2^* | 0.75 |  |  |  |

**Supplementary Table 3 Analysis of variance and broad-sense heritability for quality traits**
