# Supplementary material for: Quantitative Trait Locus (QTLs) Mapping for Quality Traits of Wheat Based on High Density Genetic Map Combined With Bulked Segregant Analysis RNA-seq (BSR-Seq) Indicates That the Basic 7S Globulin Gene Is Related to Falling Number
Source: Front Plant Sci. 2020 Dec 10;11:600788. doi: 10.3389/fpls.2020.600788 (PMC7793810; doi:10.3389/fpls.2020.600788)
Supplement: Supplementary Figure 1 — Frequency distribution of quality traits in the RILs of Chuanmai 42 × Chuanmai 39 in three environments. [file Data_Sheet_1.zip › Table S4.DOCX]

| **Sample** | **Q30 (%)** | **GC (%)** | **SLAF** | **SNP** | **Homo-SNP** | **Aver Depth** |
| --- | --- | --- | --- | --- | --- | --- |
| Chuanmai 39 | 90.59 | 43.8 | 345,812 | 857,257 | 835,041 | 31.95 |
| Chuanmai 42 | 90.62 | 43.76 | 366,633 | 1,092,628 | 1,061,671 | 57.33 |
| progeny | 90.62 | 43.55 | 339,407 | 739,557 | 719,089 | 11.62 |

**Supplementary Table 4 High quality data and SNPs generated by sequencing the SLAF library**
