# Supplementary material for: Quantitative Trait Locus (QTLs) Mapping for Quality Traits of Wheat Based on High Density Genetic Map Combined With Bulked Segregant Analysis RNA-seq (BSR-Seq) Indicates That the Basic 7S Globulin Gene Is Related to Falling Number
Source: Front Plant Sci. 2020 Dec 10;11:600788. doi: 10.3389/fpls.2020.600788 (PMC7793810; doi:10.3389/fpls.2020.600788)
Supplement: Supplementary Figure 1 — Frequency distribution of quality traits in the RILs of Chuanmai 42 × Chuanmai 39 in three environments. [file Data_Sheet_1.zip › Table S5.DOCX]

| **Chromosome** | **Total** | **Total** | **Average** | **Max** | **Gap** |
| --- | --- | --- | --- | --- | --- |
|  | **Marker** | **Distance (cM)** | **Distance (cM)** | **Gap (cM)** | **< 5 cM(%)** |
| 1A | 1258 | 99.12 | 0.08 | 5.13 | 99.84 |
| 2A | 260 | 109.94 | 0.42 | 9.15 | 99.23 |
| 3A | 312 | 118.32 | 0.38 | 4.36 | 100.00 |
| 4A | 263 | 108.76 | 0.41 | 13.28 | 98.09 |
| 5A | 650 | 181.88 | 0.28 | 5.45 | 99.85 |
| 6A | 298 | 98.78 | 0.33 | 7.81 | 98.99 |
| 7A | 718 | 143.09 | 0.20 | 8. 11 | 99.86 |
| A genome | 3759 | 859.89 | 0.30 | 13.28 | 99.41 |
| 1B | 1901 | 86.01 | 0.05 | 4.56 | 100.00 |
| 2B | 859 | 150.91 | 0.18 | 3.60 | 100.00 |
| 3B | 1513 | 110.8 | 0.07 | 6.10 | 99.87 |
| 4B | 787 | 196.21 | 0.25 | 7.80 | 99.75 |
| 5B | 774 | 173.88 | 0.22 | 12.86 | 99.74 |
| 6B | 575 | 124.82 | 0.22 | 4.72 | 100.00 |
| 7B | 478 | 180.24 | 0.38 | 11.07 | 99.37 |
| B genome | 6887 | 1022.87 | 0.20 | 12.86 | 99.82 |
| 1D | 135 | 150.06 | 1.11 | 8.76 | 97.01 |
| 2D | 271 | 141.02 | 0.52 | 14.63 | 99.63 |
| 3D | 751 | 175.36 | 0.23 | 4.53 | 100.00 |
| 4D | 371 | 96.92 | 0.26 | 18.13 | 99.73 |
| 5D | 191 | 106.64 | 0.56 | 7.15 | 98.95 |
| 6D | 189 | 198.96 | 1.05 | 15.45 | 94.15 |
| 7D | 120 | 108.22 | 0.90 | 8.90 | 93.28 |
| D genome | 2028 | 977.18 | 0.66 | 18.13 | 97.54 |
| Total | 12674 | 2859.94 | 0.23 | 18.13 | 93.28 |

**Supplementary Table 5 Characteristics of the 21 chromosomes of the wheat genetic map**
