# Supplementary material for: Quantitative Trait Locus (QTLs) Mapping for Quality Traits of Wheat Based on High Density Genetic Map Combined With Bulked Segregant Analysis RNA-seq (BSR-Seq) Indicates That the Basic 7S Globulin Gene Is Related to Falling Number
Source: Front Plant Sci. 2020 Dec 10;11:600788. doi: 10.3389/fpls.2020.600788 (PMC7793810; doi:10.3389/fpls.2020.600788)
Supplement: Supplementary Figure 1 — Frequency distribution of quality traits in the RILs of Chuanmai 42 × Chuanmai 39 in three environments. [file Data_Sheet_1.zip › Table S6.DOCX]

| **Chromosome** | **Spearman correlation coefficient** |
| --- | --- |
| 1A | 0.9 |
| 1B | 0.94 |
| 1D | 0.98 |
| 2A | 0.97 |
| 2B | 0.99 |
| 2D | 0.88 |
| 3A | 0.98 |
| 3B | 0.85 |
| 3D | 1 |
| 4A | 0.97 |
| 4B | 0.98 |
| 4D | 0.99 |
| 5A | 0.99 |
| 5B | 0.99 |
| 5D | 0.95 |
| 6A | 0.99 |
| 6B | 0.98 |
| 6D | 0.96 |
| 7A | 0.99 |
| 7B | 0.98 |
| 7D | 0.99 |

**Supplementary Table 6 Spearman correlation coefficient of Genetic and physical position**
