# Supplementary material for: Quantitative Trait Locus (QTLs) Mapping for Quality Traits of Wheat Based on High Density Genetic Map Combined With Bulked Segregant Analysis RNA-seq (BSR-Seq) Indicates That the Basic 7S Globulin Gene Is Related to Falling Number
Source: Front Plant Sci. 2020 Dec 10;11:600788. doi: 10.3389/fpls.2020.600788 (PMC7793810; doi:10.3389/fpls.2020.600788)
Supplement: Supplementary Figure 1 — Frequency distribution of quality traits in the RILs of Chuanmai 42 × Chuanmai 39 in three environments. [file Data_Sheet_1.zip › Table S7.DOCX]

| **QTL** | **Chr** | **Position (cM)** | **Physical distance（bp）** | **Marker interval** | **LOD** | **Additive effect** | **PVE（%）** | **Env.** |
| --- | --- | --- | --- | --- | --- | --- | --- | --- |
| **FN** | | | | | | | | |
| QFN.cib-5D.1 | 5D | 0 | 144933~3154627 | M85128~M85140 | 9.54 | -68.33 | 22.93 | E1 |
|  |  | 0~3.62 | 144933~4897157 | M85128~M85146 | 9.36 | -24.02 | 14.31 | BLUP |
| QFN.cib-1A | 1A | 83.47~83.83 | 548851388~572328430 | M7809~M8072 | 3.63~4.05 | -21.87~-22.2 | 6.32~6.52 | E2 |
| QFN.cib-2A | 2A | 100.83~101.67 | 757691505~758507321 | M27947~M27973 | 4.83~5.48 | -27.24~-28.79 | 9.82~10.96 | E2 |
| QFN.cib-4D | 4D | 89.56~94.53 | 499079449~500990394 | M69007~M69017 | 3.02~3.58 | -14.68~-16.15 | 2.85~3.45 | E2 |
| QFN.cib-5D.2 | 5D | 18.18~19.91 | 44050236~48538350 | M85261~M85277 | 3.04~3.54 | -16.39~-19.81 | 3.55~5.19 | E2 |
| **GPC** | | | | | | | | |
| QGPC.cib-1D | 1D | 82.85~83.31 | 321609330~326118348 | M23060~M23076 | 4.33~4.34 | -0.45 | 7.21~7.22 | E1 |
| QGPC.cib-3D | 3D | 152.15~152.62 | 579402020~580419655 | M56139~M56153 | 3.17~3.49 | -0.36~-0.37 | 8.37~9.08 | E2 |
| QGPC.cib-2D | 2D | 32.81~33.18 | 59491933~63553895 | M35619~M35536 | 4.25~5.44 | -0.42~-0.45 | 9.79~11.16 | E3 |
| QGPC.cib-4A.1 | 4A | 7.03~7.29 | 11740277~11769028 | M56444~M56443 | 3.64~3.72 | -0.35~-0.36 | 6.87~7.05 | E3 |
| **PV** | | | | | | | | |
| QPV.cib-3B.1 | 3B | 23.86~24.12 | 144818391~162247972 | M42591~M42778 | 4.67~5.09 | 16.21~16.6 | 10~10.49 | E1 |
| QPV.cib-3B.2 | 3B | 38.62~38.88 | 416511028~495084639 | M50136~M50542 | 3.04 | 20.36 | 7.23 | E2 |
| QPV.cib-4D | 4D | 96.09~96.65 | 502685898~505461931 | M69071~M69046 | 3.28~3.68 | -19.13~-19.79 | 6.39~6.84 | E2 |
| QPV.cib-2A | 2A | 55.34~55.96 | 718649359~718649784 | M27551~M27554 | 3.21~3.27 | 19.51~19.66 | 6.9~7.01 | E3 |
| QPV.cib-5D | 5D | 0 | 144933~3154627 | M85128~M85140 | 3.23 | 19.33 | 6.78 | E3 |
| **TV** | | | | | | | | |
| QTV.cib-2D | 2D | 31.14~31.55 | 64998545~68308371 | M35768~M35745 | 3.65 | -11.91 | 1.89 | E2 |
| QTV.cib-3D | 3D | 84.46~84.92 | 521546201~521717215 | M55248~M55237 | 3.13~3.31 | 23.04~23.25 | 8.01~8.15 | E3 |
| **PT** | | | | | | | | |
| QPT.cib-2A | 2A | 95.17~97.18 | 755286661~755935207 | M27887~M27898 | 4.31~7.63 | 0~0.04 | 0~2.23 | E1 |
| **BD** | | | | | | | | |
| QBD.cib-1A | 1A | 24.43~24.69 | 304124798~320009333 | M5332~M5465 | 8.09~8.22 | -28.46~-28.55 | 17.27~17.38 | E1 |
| QBD.cib-2D | 2D | 121.58~122.43 | 629280415~631638630 | M37018~M37034 | 3.11~3.22 | -16.05~-16.49 | 5.49~5.8 | E1 |
| QBD.cib-3B | 3B | 27.23~27.7 | 191474131~256262712 | M43577~M45210 | 5.89~6.59 | 21.45~22.66 | 9.81~10.95 | E1 |
| QBD.cib-7A | 7A | 83.33~83.59 | 522340394~538292722 | M103479~M103823 | 3.13~3.3 | -16.94~-18.04 | 6.12~6.94 | E1 |
| QBD.cib-5D | 5D | 46.3~49.92 | 242423747~265994623 | M85878~M85834 | 3.2~5.51 | 10.85~14.95 | 4.13~7.83 | E2 |
| QBD.cib-5B | 5B | 140.75~143.63 | 612726183~634376558 | M84001~M84281 | 3.35~7.31 | 5.79~11.22 | 2~7.5 | E3 |
| **SB** | | | | | | | | |
| QSB.cib-1A | 1A | 24.43~24.69 | 304124798~320009333 | M5332~M5465 | 3.37~3.51 | -32.94~-33.51 | 9.93~10.28 | E1 |
| QSB.cib-2B | 2B | 10.42~10.93 | 48081152~52240905 | M28709~M28766 | 5.22~5.45 | 21.84~22.22 | 10.73~11.1 | E3 |

**Supplementary Table 7 Additive QTL characterization for quality traits in RILs of wheat in single environments**
