# Supplementary material for: Quantitative Trait Locus (QTLs) Mapping for Quality Traits of Wheat Based on High Density Genetic Map Combined With Bulked Segregant Analysis RNA-seq (BSR-Seq) Indicates That the Basic 7S Globulin Gene Is Related to Falling Number
Source: Front Plant Sci. 2020 Dec 10;11:600788. doi: 10.3389/fpls.2020.600788 (PMC7793810; doi:10.3389/fpls.2020.600788)
Supplement: Supplementary Figure 1 — Frequency distribution of quality traits in the RILs of Chuanmai 42 × Chuanmai 39 in three environments. [file Data_Sheet_1.zip › Table S8.DOCX]

| **Supplementary Table 8 FN of eight genotypes in different environments*** | | | | | | | | |
| --- | --- | --- | --- | --- | --- | --- | --- | --- |
|  | *QFN.cib-3A+QFN.cib-2B+QFN.cib-3D* | *QFN.cib-3A+QFN.cib-3D* | *QFN.cib-2B+ QFN.cib-3D* | *QFN.cib-2B+ QFN.cib-3A* | *QFN.cib-3D* | *QFN.cib-3A* | *QFN.cib-2B* | null |
| E1 | 583.24±150.88cd | 520.36±101.05abcd | 477.5±61.31abc | 621.83±193.87d | 463.75±153.56ab | 573.66±124.79bcd | 478.63±73.69abc | 442.28±138.4a |
| E2 | 452.48±83.12e | 390.72±91.72de | 401.63±96.29de | 368.7±97.78d | 350.22±110.84cd | 291.08±88.14bc | 266.67±65.82ab | 216.48±72.75a |
| E3 | 375.13±51.45d | 342.11±56.07cd | 330.14±54.14cd | 312.7±74.2c | 306.06±71.87c | 240.58±79.2b | 248.6±62.21b | 194.09±64.66a |
| *Variance analysis was applied in E1, E2 and E3, respectively. The same superscripts denoted not significant difference (P>0.05), different superscripts denoted significant difference (*P*<0.05). | | | | | | | | |
