# Supplementary material for: Quantitative Trait Locus (QTLs) Mapping for Quality Traits of Wheat Based on High Density Genetic Map Combined With Bulked Segregant Analysis RNA-seq (BSR-Seq) Indicates That the Basic 7S Globulin Gene Is Related to Falling Number
Source: Front Plant Sci. 2020 Dec 10;11:600788. doi: 10.3389/fpls.2020.600788 (PMC7793810; doi:10.3389/fpls.2020.600788)
Supplement: Supplementary Figure 1 — Frequency distribution of quality traits in the RILs of Chuanmai 42 × Chuanmai 39 in three environments. [file Data_Sheet_1.zip › Table S9.DOCX]

|  | **Chuanmai 39** | **Chuanmai 42** | **LFN** | **HFN** |
| --- | --- | --- | --- | --- |
| **Raw reads** | 77871896 | 79960510 | 82118742 | 96358016 |
| **Clean reads** | 75264556 | 77424320 | 80166478 | 93943174 |
| **Clean bases** | 11.29G | 11.61G | 12.02G | 14.09G |
| **Error rate(%)** | 0.02 | 0.02 | 0.02 | 0.02 |
| **Q20(%)** | 97.91 | 98.11 | 98.04 | 97.92 |
| **Q30(%)** | 94.36 | 94.75 | 94.55 | 94.39 |
| **GC content(%)** | 58.64 | 57.34 | 58.45 | 58.21 |
| **Total reads** | 75264556 | 77424320 | 80166478 | 93943174 |
| **Total mapped** | 66482058 (88.33%) | 70052050 (90.48%) | 67466730 (84.16%) | 75431462 (80.29%) |
| **Multiple mapped** | 4966479 (6.6%) | 4497652 (5.81%) | 5317216 (6.63%) | 6854464 (7.3%) |
| **Uniquely mapped** | 61515579 (81.73%) | 65554398 (84.67%) | 62149514 (77.53%) | 68576998 (73%) |
| **Reads map to '+'** | 30734277 (40.83%) | 32753574 (42.3%) | 31083181 (38.77%) | 34287680 (36.5%) |
| **Reads map to '-'** | 30781302 (40.9%) | 32800824 (42.37%) | 31066333 (38.75%) | 34289318 (36.5%) |
| **Non-splice reads** | 43425724 (57.7%) | 44370629 (57.31%) | 43392601 (54.13%) | 48451859 (51.58%) |
| **Splice reads** | 18089855 (24.04%) | 21183769 (27.36%) | 18756913 (23.4%) | 20125139 (21.42%) |

**Supplementary Table 9 Summary of BSA sequencing and mapping**
